# Genomic profile analysis of diffuse-type gastric cancers

**DOI:** 10.1186/gb-2014-15-4-r55

**Published:** 2014-04-01

**Authors:** Yeon-Su Lee, Yun Sung Cho, Geon Kook Lee, Sunghoon Lee, Young-Woo Kim, Sungwoong Jho, Hak-Min Kim, Seung-Hyun Hong, Jung-Ah Hwang, Sook-young Kim, Dongwan Hong, Il Ju Choi, Byung Chul Kim, Byoung-Chul Kim, Chul Hong Kim, Hansol Choi, Youngju Kim, Kyung Wook Kim, Gu Kong, Hyung Lae Kim, Jong Bhak, Seung Hoon Lee, Jin Soo Lee

**Affiliations:** 1 Cancer Genomics Branch, Research Institute, National Cancer Center, Goyang-si, Gyeonggi-do, Republic of Korea; 2 Personal Genomics Institute, Genome Research Foundation, 443-270 Suwon, Republic of Korea; 3 Department of Pathology and Tumor Tissue Bank, National Cancer Center, Goyang-si, Gyeonggi-do, Republic of Korea; 4 Gastric Cancer Branch, Research Institute and Hospital, National Cancer Center, Goyang-si, Gyeonggi-do, Republic of Korea; 5 Theragen BiO Institute, TheragenEtex, 443-270 Suwon, Republic of Korea; 6 Department of Pathology, College of Medicine, Hanyang University, Seoul, Republic of Korea; 7 Department of Biochemistry, School of Medicine, Ewha Womans University, Seoul, Republic of Korea; 8 Program in Nano Science and Technology, Department of Transdisciplinary Studies, Seoul National University, Suwon 443-270, Republic of Korea; 9 Advanced Institutes of Convergence Technology Nano Science and Technology, Suwon 443-270, Republic of Korea; 10 Research Institute and Hospital, National Cancer Center, Goyang-si, Gyeonggi-do, Republic of Korea

## Abstract

**Background:**

Stomach cancer is the third deadliest among all cancers worldwide. Although incidence of the intestinal-type gastric cancer has decreased, the incidence of diffuse-type is still increasing and its progression is notoriously aggressive. There is insufficient information on genome variations of diffuse-type gastric cancer because its cells are usually mixed with normal cells, and this low cellularity has made it difficult to analyze the genome.

**Results:**

We analyze whole genomes and corresponding exomes of diffuse-type gastric cancer, using matched tumor and normal samples from 14 diffuse-type and five intestinal-type gastric cancer patients. Somatic variations found in the diffuse-type gastric cancer are compared to those of the intestinal-type and to previously reported variants. We determine the average exonic somatic mutation rate of the two types. We find associated candidate driver genes, and identify seven novel somatic mutations in *CDH1*, which is a well-known gastric cancer-associated gene. Three-dimensional structure analysis of the mutated E-cadherin protein suggests that these new somatic mutations could cause significant functional perturbations of critical calcium-binding sites in the EC1-2 junction. Chromosomal instability analysis shows that the *MDM2* gene is amplified. After thorough structural analysis, a novel fusion gene *TSC2*-*RNF216* is identified, which may simultaneously disrupt tumor-suppressive pathways and activate tumorigenesis.

**Conclusions:**

We report the genomic profile of diffuse-type gastric cancers including new somatic variations, a novel fusion gene, and amplification and deletion of certain chromosomal regions that contain oncogenes and tumor suppressors.

## Background

Stomach cancer ranks as the third most important cause of global cancer mortality [1]. Histopathologically, gastric cancer (GC) can be classified into two categories based on morphological differences: intestinal-type GC (IGC) and diffuse-type GC (DGC) [2,3]. IGC is typically associated with *Helicobacter pylori* infection, and is especially common in Japan and Korea [4-6]. DGC is uniformly distributed geographically, and includes aggressive clinical forms, such as linitis plastica, which have poor prognosis, especially in young patients [7,8]. Genomic DNA modifications leading to GC can happen as a result of several environmental risk factors such as a high-salt diet and tobacco smoking [9]. Although the incidence of IGC has decreased steadily over several decades (44% reduction from 1978 to 2005), DGC increased rapidly (by 62%) from 1978 up to 2000, before decreasing slightly in 2001–2005 [10]. Despite the cumulative evidence that IGC and DGC develop via different carcinogenic pathways [11,12], detailed genomic scale data for DGC are lacking because of limited availability of clinical samples and a low level of purity of the cancer cell population.

To date, very few genes associated with GC subtypes have been identified. The *CDH1* gene, which encodes the E-cadherin protein, are the best-known genes associated with hereditary DGC (HDGC) [13-16]. Genetic screening for these mutations has been suggested in order to diagnose early-onset GC [17]. E-cadherin dysfunction, caused by mutations, loss of heterozygosity, and promoter hypermethylation, is the most well-established defect in GC initiation and development [18-20]. A genome-wide association study showed that polymorphisms in the prostate stem cell antigen gene (*PSCA*) are strongly associated with susceptibility to DGC [21]. The microarray-based method, however, is limited to single nucleotide variations, and cannot detect copy-neutral structural variations (SVs). Two recent studies reported on GC exomes, and showed that mutations in the *ARID1A* gene are frequently detected in GC with microsatellite instability, and in Epstein-Barr virus (EBV)-positive GCs [22,23]. No analysis of GC subtypes was performed, and the majority of the samples analyzed in the studies were from patients with IGC.

Next-generation sequencing (NGS) has allowed researchers to detect disease-associated variations, and helped uncover the underlying mechanisms of disease development. In particular, whole genome sequencing (WGS) can detect most genomic variations, including SVs, such as intrachromosomal and interchromosomal rearrangements. Alternatively, whole exome sequencing (WES), a captured-target sequencing method, can be used for high-depth sequencing of a large number of samples at a relatively low cost [24], although only single nucleotide variations (SNVs) and small insertions or deletions (indels) can be identified using this method. WGS and WES each have advantages and disadvantages, and a number of recent studies have used both methods [25-27].

Here we present detailed characterization of DGC genomes from matched tumor and normal samples by generating whole genomic profiles followed by WES. We used blood samples as a normal control, as in previous studies [28-31]. In order to find DGC-specific variations, IGC genomes were also analyzed and compared with variations identified in genomes of DGCs. Three-dimensional protein structure analysis was performed for novel somatic mutations of the *CDH1* gene, and this identified critical regions that were functionally altered by the mutations. In addition, we found a novel fusion gene that could be involved in tumorigenesis.

## Results and discussion

### Whole genome and exome sequencing

Tumor and matched normal (blood) samples from 14 patients with DGC (the clinicopathological characteristics of these patients are shown in Table S1 in Additional file 1), who were all relatively young (median age 38 years) Korean women, were sequenced using an Illumina HiSeq 2000, which produced paired-end, 90-base and 101-base DNA reads. Additionally, five pairs of tumor and matched normal samples from patients with IGC (median age 42 years) were subjected to DNA sequencing; one of these samples was identified later as a case of microsatellite instability (MSI) and hence was excluded from the mutation analysis. None of the samples had any familial history of cancer, and the subtypes were histopathologically confirmed. Only tumor cells were collected by macrodissection after hematoxylin staining.

For the whole genome analysis, on average, 92 gigabases (Gb) per sample were produced at approximately 32 times sequencing depth, reaching 3.5 terabases (Tb) in total, and were mapped to the reference genome (NCBI build 37, hg19) at a mapping rate greater than 94.5% (for sequencing statistics, see Additional file 1: Table S2). Using the final 3.3 Tb of mapped reads, a genomic profile database was constructed for detecting SNVs, copy number variations (CNVs), and SVs. Because the cellular purity of a tumor sample is a critical feature in cancer genome analysis, it was evaluated using an in-house calculation method (see Materials and Methods; see Additional file 1: Table S3 and Figure S1). Although we tried to collect only tumor cells, our samples still showed a high level of stromal admixture. To increase the accuracy of mutation detection in genic regions even in low-purity samples, additional WES was performed at approximately 103 times sequencing depth on average, which produced a total of 17 Gb sequence data. The captured WES covered 93.1% of the genic region at 10 times or greater depth, and this coverage is similar to that of previously reported exome data on GC [22,23].

**Figure 1:**
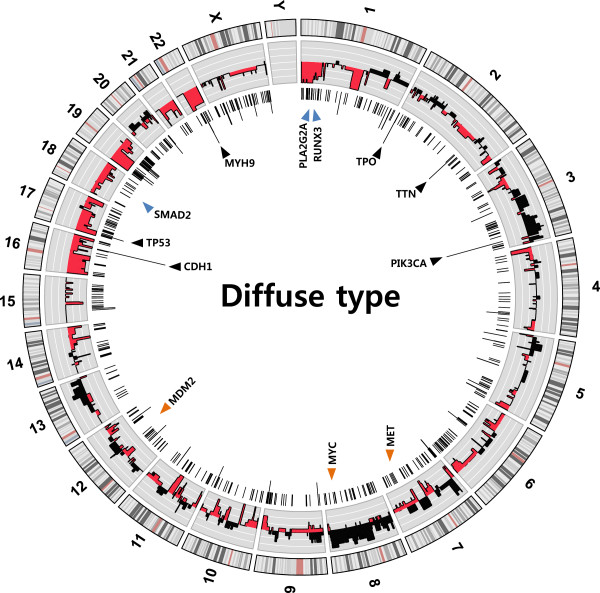
**Whole genome distribution of somatic mutations and duplication or deletion events in diffuse-type gastric cancers (DGCs).** All the somatic mutations, including duplication/deletion events, which were found in the 14 DGC genomes, are merged in the circus plot. From outside to inside, the plot presents the following characteristics: chromosome ideograms, frequency of cumulative amplification or deletion events (black, amplification; red, deletion), and the number of somatic non-synonymous single nucleotide variations (nsSNVs), indels, and SNVs in splice sites for each gene. Black triangles indicate highly mutated genes. Orange triangles denote oncogenes, and blue triangles indicate the tumor suppressors.

Combining the WGS and WES data, we detected somatic alterations in the DGC samples, and compared them with the IGC alterations (the data are summarized in Figure 1 as a circus diagram). To verify our data, we combined and analyzed them with previously reported exome data from two different studies (24 IGC and 5 DGC samples, not including MSI and mixed samples) [22,23] and from array comparative genome hybridization (CGH) data (16 IGC and 14 DGC samples) [32]. Although those studies used mainly IGCs and included only a small number of DGC samples, they could be complementary to our data as a control (by providing an increased number of IGC data and elimination of tissue specificity). In the combined dataset, we compared the differences in alterations between the DGC and IGC samples.

### Identification of diffuse-type-specific SNVs and indels

In each sample pair, we identified approximately 3.7 million SNVs, which were verified using single nucleotide polymorphism (SNP) chips (average concordance rate: 99.2%; see Additional file 1: Table S4), and approximately 0.69 million indels (for details, see Additional file 1: Table S5 and Table S6). We first assessed mutational frequency of both types of GC at the single nucleotide level (see Additional file 1: Figure S2 a, b). The somatic mutation spectrum was dominated by C > T (G > A) transitions in both the DGC and IGC samples, and there were no significant differences in mutational contexts between the two GC types, in accordance with previous studies of GC [23,30]. When we analyzed two previously reported exome datasets, we found that the spectrum of the nucleotide substitution ratio was similar to our data (see Additional file 1: Figure S2c, d).

Although the mutation spectrum of DGC is similar to that of IGC, individual mutations in affected genes were different. By subtracting mutations found in normal blood genomes, we identified 922 non-synonymous SNVs (nsSNVs) as somatic mutations in the 18 tumor samples (see Additional file 1: Table S7; see Additional file 2). The average mutation rate of the 18 GCs (1.97 mutations/Mb) was comparable with that reported in other studies on colon, pancreatic, and liver cancers [33-35]. Of 847 mutated genes affected by the 922 nsSNVs, 581 were in 14 DGC cases, 288 were in 4 IGC cases, and 22 (2.6%) were common to both types. The MSI sample, which was excluded from the comparative analysis, showed approximately six times more SNVs and indels than did the other samples; this result is in agreement with a previous report [22]. When we combined the two previously reported exome datasets, we identified 967 and 2,077 somatic nsSNVs in 19 DGCs and 28 IGCs, respectively. The somatic mutation rate of the IGCs (3.71 mutations/Mb in the 28 samples) was higher than that of the DGCs (2.29 mutations/Mb in the 19 samples) (see Additional file 1: Table S8). Previously published research suggests that melanoma and lung cancer have high mutation rates, owing to the involvement of potent mutagens [36]. Likewise, it is possible that IGC has this high mutation rate because its tumorigenic mechanism may be associated more with environmental and/or parasitic mutagens compared with DGC.

For individual variations, putative cancer-causative genes were predicted by driver gene score calculation (see Additional file 1: Table 1 and Table S9). The *CDH1* gene was found to be abundantly mutated in DGC (*P* = 1.29 × 10^−2^), including six somatic mutations (three missense, one nonsense, one frameshift, and one splice site mutations) that have not been reported previously, whereas only one missense mutation was found in the IGC samples (Table 2). All seven *CDH1* somatic mutations were verified by Sanger sequencing (see Additional file 1: Table S10 and Table S11). In our DGC samples, 35.7% (5/14) had *CDH1* somatic mutations, and it has been reported that the frequencies of *CDH1* somatic mutations in sporadic DGCs can vary from 3% to greater than 50% [19,37-40]. It was verified that in countries with a high incidence of sporadic GC (such as Japan and Korea), the frequency of germline mutations in familial GCs is low compared with that in low-incidence countries [41,42]. Therefore, we speculate that the overall GC incidence is also related to the frequency of *CDH1* somatic mutations. Additionally, one germline mutation (T340A) in *CDH1* was found in both tumor and corresponding blood genomes from two samples (D-14, DGC; M-01, MSI-type). Although T340A is a causative mutation in HDGC [43], these two patients did not have any familial history such as GC or lobular breast cancer. Two previous reports analyzing exome data of GC did not identify *CDH1* as a highly ranked gene (only one missense mutation in an MSI IGC sample) [22,23]. This discrepancy may be due to the small number of samples of DGC in those studies (2 out of 22 and 3 out of 15 samples were DGCs, respectively). In the present work, *PIK3CA* and *TP53*, well-known cancer-associated genes, were the most frequently mutated genes in both DGC and IGC see Table 1 and Table S9 in Additional file 1. Mutations in two known *PIK3CA* hotspots (E545K and H1047L) were found in four DGC samples. Additionally, one nsSNV mutation (Q546K) adjacent to the E545K mutation was found in one DGC sample. In total, 5 out of 14 DGC samples (approximately 30%) harbored nsSNVs in *PIK3CA*, which is an oncogene whose mutated form exhibits increased kinase activity, causing cancer cell proliferation [44]. We then compared the low frequency (16–17%) of the nsSNVs in *PIK3CA* in reports by others [22,23,44] (who mostly used IGC samples) and the results of our combined analysis (31.5% for DGC, 14.3% for IGC) (see Additional file 1: Table S9). It appears that the relatively high mutation rates of *PIK3CA* in DGC may reflect the specificity of mutations in this gene to this type of cancer. Additionally, three samples (two DGC and one IGC) contained both nsSNV and a copy loss of *TP53*, indicating a homozygous loss of function in *TP53*, as previously reported [45]. An SNP in the *PSCA* gene (rs2976329) has been reported to be associated with increased risk of DGC in Japanese and Korean populations [21]. This SNP was also enriched in the majority of DGC samples in our study, (9 out of 14 patients), indicating that our analyzed samples represent typical patients with DGC in East Asia. Additionally, a nonsense mutation (R1446*) in the *ARID1A* gene, was found in one DGC sample (D-08). Although mutations in *ARID1A* are frequently detected in MSI and in EBV-positive GCs [22,23], the D-08 sample showed no EBV infection, and an MSI sample (M-01) did not have any *ARID1A* gene mutations either. From variations in candidate driver genes, 88 nsSNVs, 4 small indels, and 2 SNVs in a splice site were verified using conventional Sanger sequencing. Seven of these mutations could not be tested because of PCR failure, and of the remaining 87 mutations, 96.6% were confirmed as true somatic mutations (see Additional file 1: Table S10 and Table S11).

**Table 1:** Top candidate driver genes in 14 diffuse-type gastric cancers

**Gene**	**Samples, n**	**nsSNVs, n**	**SNVs in splice site, n**	**Indels, n**	***P*-value**	**Driver gene score**
*PIK3CA*	5	5	0	0	3.63 × 10^−12^	9.83
*CDH1*	5	4	1	1	4.64 × 10^−10^	8.02
*SNRPN*	2	2	0	0	1.86 × 10^−07^	5.60
*TP53*	2	2	0	0	4.88 × 10^−07^	5.36
*CMKLR1*	2	2	0	0	5.33 × 10^−07^	5.36
*CYP2A7*	2	2	0	0	1.53 × 10^−06^	4.99
*GUCY1B3*	2	2	0	0	1.97 × 10^−06^	4.99
*PAPOLB*	2	2	0	0	2.15 × 10^−06^	4.99
*MYH9*	3	3	0	0	2.27 × 10^−06^	4.99
*FAM71B*	1	2	0	0	2.51 × 10^−06^	4.99
*C10orf90*	2	2	0	0	3.76 × 10^−06^	4.86
*AKAP8*	2	2	0	0	4.59 × 10^−06^	4.81
*ZC3H12B*	2	2	0	0	5.87 × 10^−06^	4.74
*SFTA3*	1	1	0	0	6.86 × 10^−06^	4.70
*SENP7*	2	2	0	0	7.65 × 10^−06^	4.68
*TMPRSS6*	2	2	0	0	8.38 × 10^−06^	4.67
*PAGE2*	1	1	0	0	9.94 × 10^−06^	4.62

For additional driver gene lists, see Additional file 1: Table S9.

**Table 2:** ***CDH1 *** **alterations in 18 gastric cancers**

**Sample**	**Type**	**Alteration**	***CDH1 *** **region**
D-01 T	CNV	Loss	Exons 1 to 16
D-02 T	SNV	N256S	Exon 6
	CNV	Loss	Exons 1 to 16
D-03 T	SNV	Splice site	Donor site of Intron 4
D-04 T	CNV	Loss	Exons 1 to 16
D-05 T	SNV	D257N	Exon 6
	INS	S829fs	Exon 16
D-09 T	SNV	V252G	Exon 6
	SV	Break point	Intron 2
D-10 T	CNV	Loss	Exons 1 to 16
D-11 T	CNV	Loss	Exons 1 to 16
D-12 T	SNV	Q23*	Exon 2
D-13 T	CNV	Loss	Exons 1 to 16
	SV	Break point	Introns 2 and 10
D-14 T	SV	Break point	Introns 2, 5 and 9
I-01 T	CNV	Loss	Exons 1 to 16
I-02 T	CNV	Loss	Exons 1 to 16
I-03 T	SNV	D221G	Exon 5
	SV	Break point	Introns 10 and 13
I-04 T	CNV	Loss	Exons 1 to 16

CNV, copy number variation; INS, small insertion; SNV, single nucleotide variation; SV, structural variation.

The somatic variations were then mapped onto the Kyoto Encyclopedia of Genes and Genomes (KEGG) pathways database. This analysis revealed that the mutated genes of DGCs were significantly associated with the calcium signaling pathway (*P* = 7.00 × 10^−5^; see Additional file 1: Table S12 and Table S13). Low calcium intake may contribute to GC development [46]. Calcium is essential for the function of E-cadherin, and a loss of E-cadherin-mediated adhesion is involved in the transition from a benign lesion to invasive metastatic cancer [47]. Furthermore, the somatic mutations were strongly associated with pathways related to small cell lung cancer (*P* = 1.00 × 10^−6^ in DGC and *P* = 4.24 × 10^−2^ in IGC). In particular, genes involved in focal adhesion pathways, such as *ITGA*, *PIK3CA*, and *PTEN*, were frequently mutated.

### SV and CNV analysis

SVs were detected based on discordantly mapped read pairs, and any SVs that were present in the patients’ germline genomes were excluded. On average, we found 552 somatic SVs per DGC sample pair (211 large insertions, 264 large deletions, 27 inversions, 44 intrachromosomal translocations, and 6 interchromosomal translocations). We found 664 somatic SVs in each IGC sample pair (285 large insertions, 283 large deletions, 34 inversions, 38 intrachromosomal translocations, and 24 interchromosomal translocations) (for details for each sample, see Additional file 1: Table S14 and Figure S3). Additionally, we found 2,258 genes to be impaired, and 1,736 of these were found only in the DGC samples (for data for each sample, see Additional file 1: Table S15; and see Additional file 3). Three tumor suppressor genes *FHIT*, *WWOX*, and *MIPOL1*, which were reported in a previous GC study [30], had impairments due to the SVs (*FHIT* in 11 samples, *WWOX* in 5 samples, and *MIPOL1* in 3 samples).

**Figure 2:**
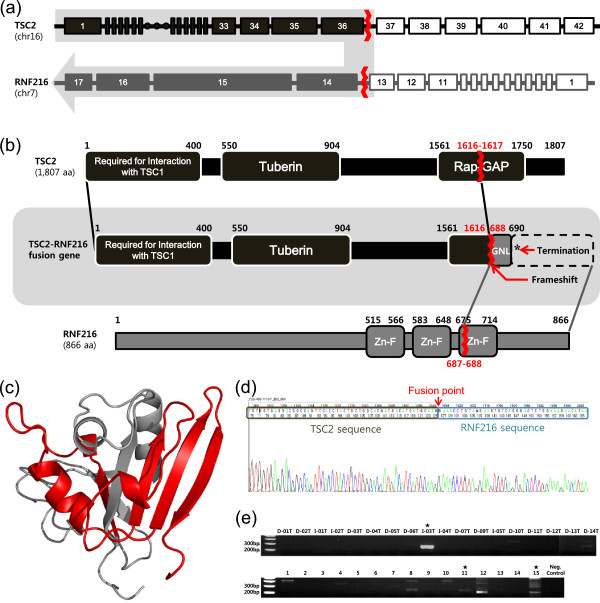
***TSC2*-*RNF216 *fusion gene breakage. (a)** Exon structure of the *TSC2*-*RNF216* fusion gene. The numbers in the boxes are the exon numbers of each gene. Red lines indicate the fusion points. **(b)** Protein domain structure of the TSC2-RNF216 fusion protein. The Rap-GAP domain of TSC2 was broken, and RNF216 had a frameshift mutation causing premature termination by the interchromosomal rearrangement. **(c)** Structure of the TSC2 Rap-GAP domain. The red region is the remaining Rap-GAP domain region, and the gray region is the Rap-GAP domain that is deleted in the *TSC2*-*RNF216* fusion gene. **(d)** RNA sequence of the *TSC2*-*RNF216* fusion gene. Position 136 is shown as N. Either an A or G base produces a termination codon (TAA or TAG). **(e)** Verification of the *TSC2*-*RNF216* fusion transcript in RNA (cDNA) by means of PCR amplification and electrophoresis.

Fusion genes generated by a chromosomal rearrangement were also analyzed, and 19 fusion gene candidates were identified (see Additional file 1: Table S16), including a novel fusion gene, *TSC2*-*RNF216*, found in one sample (Figure 2a, b). *TSC2* encoding the tuberin protein was previously suggested as a tumor suppressor gene involved in the mammalian target of rapamycin (mTOR) pathway [48,49]. In addition, *RNF216*, encoding E3 ubiquitin-protein ligase, is involved in cytokine function by preventing the sustained activation of nuclear factor (NF)-κB [50]. The Rap GTPase activating protein (Rap-GAP) domain of the TSC2 protein, which is related to the intrinsic GTPase activity of the Ras-related proteins RAP1A and RAB5, was broken by this chromosomal translocation (Figure 2c). In addition, the zinc finger domains of the RNF216 protein were not expressed in the fusion gene, because of a frameshift that caused premature termination. Using reverse transcription polymerase chain reaction (RT-PCR) followed by sequencing analysis, the expression of this fusion gene in the patient’s tissue was confirmed. After testing an additional set of 15 GC patient tissues, we identified 2 patients expressing the fusion gene (Figure 2d, e). This chromosomal translocation can lead to altered cellular behavior both by disrupting the normal functioning of the gene and causing expression of the fusion gene product, which may compete against the normal gene. The fusion gene can competitively interfere with tumor suppressor pathways and activate NF-κB-mediated cytokine signaling.

In DGCs, chromosomes 16, 17, 19, 20, 21, and 22 contained an increased amount of block deletions, while chromosomes 3, 7, 8, and 13 showed notably increased duplications (Figure 1). Many tumor suppressor genes, such as *CDH1*, *PLA2G2A, RUNX3*, *SMAD2*, and *TP53*, are located in extensively deleted chromosomal regions. Notably, somatic mutation (nsSNV or splice site mutation) and copy number loss of *CDH1* were generally mutually exclusive: four out of five DGC samples with somatic mutation did not have gene copy number losses, and eight out of nine DGC samples with a *CDH1* gene copy number loss did not have any somatic mutations in *CDH1*. Only one sample (1/18, 5.6%) had both alterations (mutation and copy number loss) concomitantly, and this observation coincides with previous studies reporting that concomitant alterations in *CDH1* are rare [19,40,51,52]. When we considered SVs in *CDH1* together, we found that other three samples had a mutation/copy number loss concomitant with SV. Additionally, the copy numbers of the oncogene *MYC* were increased in five DGC samples (see Additional file 4), and copy numbers of *MET* were increased in three DGC samples [53]. The oncogenes *MOS* and *ZHX2* also showed a copy number gain in five and four DGC samples, respectively. More than half of the samples (10 out of 18) showed a copy number reduction of *ARID1A*, which is a driver gene for ovarian clear cell carcinoma, and a chromatin remodeler in GC [22,54,55]. It is known that the majority of GCs with *ARID1A* mutations show lower protein expression compared with GCs without an *ARID1A* mutation [22]. If the dosage effect is important in these cancer tissues, copy number reduction of *ARID1A* could be a possible cancer-associated factor.

**Figure 3:**
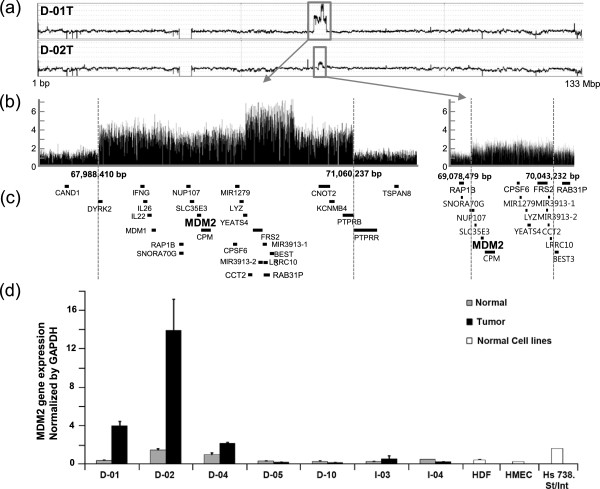
**The duplication region of the *MDM2 *gene on chromosome 12 in samples D-01 T and D-02 T. (a)** Mapping depth plots of the two chromosomes. **(b)** Thin black spikes were read at mapping depth of 2000-base width. The *y*-axis shows relative depth. Each unit represents approximately 30 times sequencing depth. **(c)** Gene positions and names around the amplified regions. The black bands show gene locations. **(d)***MDM2* transcript levels in tumor and adjacent normal tissue paired samples and normal cell lines. Quantitative RT-PCR was used to measure *MDM2* mRNA levels in samples D-01 and D-02 (containing amplified *MDM2* regions), D-04, D-05, D-10, I-03, and I-04 (without amplified *MDM2* regions), and three normal cell lines (HDF, HMEC, and Hs 738.St/Int). Error bars were calculated from two separated experiments of triplicate reactions.

A large region of chromosome 12 was amplified in three DGC genomes; of these three genomes, samples D-01 T and D-02 T showed distinctively high amplification (Figure 3a). The duplication patterns were slightly different: D-01 T had a tandem duplication of 3 Mbp, whereas D-02 T had an inverted duplication of 1 Mbp (Figure 3b, c). Part of this duplicated region encodes the murine double minute (*MDM2*) gene. It was reported that in a small dataset, the *MDM2* gene was frequently amplified [56], and that this gene is associated with several cancers [57]. *MDM2* overexpression caused by the gene amplification was experimentally confirmed using quantitative RT-PCR with the tumor and adjacent normal tissue paired samples used for NGS analyses, and normal cell lines were included for comparison (Figure 3d). *MDM2* overexpression positively correlated with the copy number analysis data. Although previously reported array CGH data [32] had relatively low resolution for CNV detection, we used those data to search for a bias in alterations of gene copy number in each histopathological type. A copy number gain of genes encoding calcium channel proteins (*CACNG6*, *CACNG7*, and *CACNG8*, *P* = 4.24 × 10^−2^) was significantly more common in DGC samples (see Additional file 1: Table S17). All integrated alteration information is shown in Additional files (see Additional file 1: Table S18 and Table S19; see Additional file 5).

### 3D structural analysis of mutated CDH1

**Figure 4:**
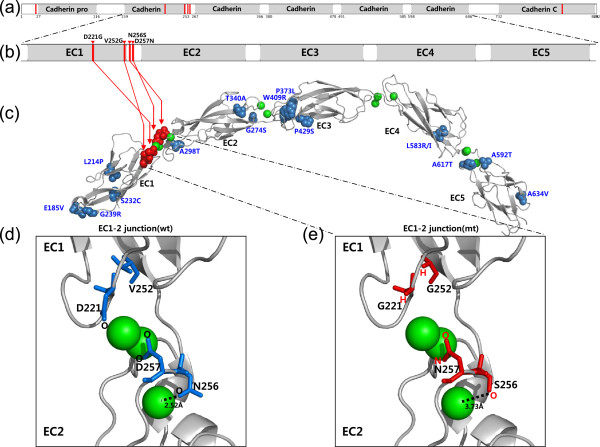
**Structure of the CDH1 protein and EC1-2 junction. (a)** The full-length E-cadherin protein has 882 amino acid residues in 7 domains. Sites of non-synonymous mutations and deletions are shown with red lines. **(b)** Red lines and triangles indicate non-synonymous mutations in extracellular cadherin (EC) domains. **(c)** CDH1 has five EC domains (EC1–EC5, which form a β-barrel structure) and four EC junctions (EC1-2, EC2-3, EC3-4, and EC4-5). The green spheres represent Ca^2+^ ions. The red and blue spheres represent somatic mutations found in this study and previously reported mutations found in hereditary diffuse-type gastric cancer, respectively. **(d, e)** CDH1 mutation sites in the EC1-2 junction. In the case of the D221G mutation, oxygen atoms of the aspartic acid side chain, which normally interact with Ca^2+^, are absent when the aspartic acid residue is replaced with a glycine. In the case of the D257N mutation, the two oxygen atoms of the Asp side chains become one oxygen atom and one nitrogen atom when aspartic acid is replaced with asparagine. In the N256S mutation, the oxygen atom of the asparagine side chain is preserved, but the distance between the oxygen atom and the Ca^2+^ ion is increased from 2.52 Å to 3.73 Å. All structures were drawn by using PyMOL Molecular Graphics System (v0.99rc6; Schrödinger LLC).

To understand how the detected mutations affect protein structure/function and activation of downstream biological pathways influencing carcinogenesis, we analyzed three-dimensional (3D) structures of the mutant E-cadherin protein found in one IGC and five DGC samples (see Additional file 2). The *CDH1* gene encodes a calcium-dependent cell adhesion glycoprotein and has five extracellular cadherin domains (EC1-EC5) (Figure 4a). It is known that the interaction between cadherin and calcium is required for dimerization, structural rigidity, and protection from proteolytic degradation [58]. Mutations in the EC1-2 and EC2-3 junctions are known to cause improper cadherin localization and diminished cell adhesion [59]. Structural analysis was carried out on four nsSNVs (D221G, V252G, N256S, and D257N), excluding a nonsense SNV (Q23*), a frameshift insertion (S829fs), and a splice site (chr16:68842472) mutation. All four nsSNVs were located in the junction between EC1 and EC2 (EC1-2 junction) (Figure 4b, c), and three nsSNVs (D221G, N256S, and D257N) were in the protein region that directly interacts with a calcium ion (Figure 4d, e). This situation could result in anomalous interactions between the cadherin domains. It is reported that A298T, D231K, and D231A mutations, which have a similar structural position at the EC1-2 junction to the somatic mutations found in this study, showed a loss of cell adhesion function [60,61]. Another nsSNV mutation, V252G, is located in the β-sheet structure of cadherin, and its side chain is oriented towards the interior. Because β-barrel structures generally contain alternating polar and hydrophobic amino acids, with the hydrophobic residues oriented toward the interior of the barrel to form a hydrophobic core, and the polar residues oriented toward the outside of the barrel on the solvent-exposed surface, the formation of the hydrophobic core may be hindered by the V252G mutation (Figure 4e). A previous exome study reported two *CDH1* mutations, P127fs (frameshift mutation in a DGC) and V694I (in an MSI IGC) [22]. Dimerization of two cadherin molecules in either a *cis* or *trans* configuration occurred at the junction between EC1-2 and EC1-2 [62], whereas mutations at the EC3-4 and EC4-5 junctions did not significantly affect cell adhesion [59]. Val694 is located in a loop region between the EC5 β-barrel and a transmembrane region distant from the EC1-2 and EC2-3 junctions. Accordingly, the V694I mutation may not be disruptive to E-cadherin protein function. Moreover, Val and Ile have a similar hydrophobic side chain and are similar in size.

Additionally, we structurally analyzed previously reported 19 missense mutations in *CDH1* (see Additional file 1; Table S20), which were found in hereditary DGC [60,63-65]. We found that the *in vitro* functional changes by the missense mutations corresponded exactly to the effects on calcium interaction and structural integrity as described above. The somatic mutations that we found were concentrated in the EC1-2 junction region, whereas the 19 germline mutations were scattered throughout the E-cadherin protein (Figure 4c). This finding coincides with previous results that germline *CDH1* mutations are not restricted to specific E-cadherin domains, but are distributed throughout all protein functional domains [66]. In this study, we identified four somatic missense mutations in exons 5 and 6, and it is known that somatic *CDH1* mutations found in sporadic DGCs cluster in exons 7 to 10 [66,67]. Exons 5 and 6 encode the structural components of EC1, EC2, and EC1-2 junction, as in the case of exons 7 and 8. Taken together, these results suggest that *CDH1* somatic mutations in the EC1-2 junction that disrupt cell adhesion function are prevalent in DGCs, and thus that dysfunction of the EC1-2 junction is specific to DGC.

## Conclusions

WGS and WES were used here to identify somatic variations that are characteristic of DGC. The samples contained both DGC and normal cells, such that the sample purity range was as low as 20% according to our genomic profile analyses. Our approach (WGS combined with exome data with sequencing depth of greater than 120 times) resulted in accurate detection of SNVs and indels in genic regions. The efficacy of this approach is evident in the verification data, which showed a positive rate of 96.6% for somatic SNVs and indels. This combination approach also has the benefit of detecting SVs and large-scale abnormalities, whereas WES alone can identify only somatic variations such as SNVs and indels in exonic regions. This strategy may facilitate analysis of heterogeneous cancer cells, an important issue in cancer genomics [68].

To the best of our knowledge, this is the first extensive genomic analysis of DGC. We identified somatic SNVs and indels in the DGC samples, compared with the IGC samples. We also found SVs and a novel fusion gene in GC samples, although their functional effects need to be validated in further studies. *CDH1* mutations are known to be prevalent in several types of cancers: gastric, colorectal, breast, thyroid, and ovarian. E-cadherin dysfunction is the most well-established defect in GC development, and our data support its importance in DGC. The DGC samples showed a high frequency of somatic mutations in *CDH1*, and protein structural analysis suggested that the mutations influence the interaction between E-cadherin and calcium, and the stability of β-barrel structures of cadherin. These results indicate that *CDH1* and the calcium signaling pathway are associated with the pathogenesis of DGC. Our data from GC genomes should improve the understanding of the mechanism via which protein structural perturbations can cause pathological changes and possibly lead to cancer development. This knowledge may help to diagnose and treat GCs in a more individualized manner, taking into account the different subtypes.

## Materials and methods

### Patients and specimen collection

This study was performed in accordance with the Declaration of Helsinki and was approved by the local ethics committee of the National Cancer Center (IRB No. NCCNCS-10-392). Signed informed consent was obtained from all participants before enrolment.

GC specimens and peripheral blood samples were collected from 18 patients (14 with DGC and 4 with IGC) who had undergone surgical resection at the National Cancer Center, South Korea, between 2005 and 2010 (see Additional file 1: Table S1). Tumor and adjacent normal specimens were examined by pathologists to remove the necrotic region and the intervening tissue, in accordance with the World Health Organization histopathological criteria. After pathological examination, the samples were snap-frozen and stored in liquid nitrogen until genomic DNA extraction.

### Nucleic acid preparation

The frozen tumor samples were macro-dissected and lightly stained with hematoxylin to identify regions consisting of 80% or more cancer cells. Genomic DNA was extracted with the MagAttract DNA Blood Midi Kit (Qiagen Inc, Valencia, CA, USA), in accordance with the manufacturer’s protocol. DNA quality was assessed using a Nanodrop spectrometer (Nanodrop Technologies, Wilmington, DE, USA). Control DNA from matched peripheral blood samples was processed in the same manner. The same frozen tumor samples were used for total RNA extraction using a Qiagen RNeasy Mini Kit (Qiagen). Quality of total RNA was assessed with Lab-on-a-Chip on an Agilent 2100 Bioanalyzer (Aglient Technologies, Santa Clara, CA, USA). The total RNA (1 μg) was used in a reverse transcription reaction with poly (dT) primers using the SuperScriptTMIII First-Strand Synthesis system (Invitrogen/Life Technologies, Grand Island, NY, USA), in accordance with the manufacturer’s instructions. DNA and RNA of adjacent normal tissues were obtained using same methods as tumor samples.

### Whole genome sequencing

Genomic DNA was sheared using Covaris S series (Covaris, MS, USA). The sheared DNA was end-repaired, A-tailed, and ligated to pair-end adapters, in accordance with the manufacturer’s protocol (Pair End Library Preparation Kit, Illumina, San Diego, CA, USA). Adapter-ligated fragments were purified and dissolved in 30 μl of elution buffer, and 1 μl of the mixture was used as a template for 12 cycles of PCR amplification. The PCR product was gel-purified using the QIAquick Gel Extraction Kit (Qiagen). Library quality and concentration were determined using an Agilent 2100 BioAnalyzer (Agilent). Libraries were quantified using a SYBR green qPCR protocol on a LightCycler 480 (Roche, Indianapolis, IN, USA), in accordance with Illumina’s library quantification protocol. Based on the qPCR quantification, libraries were normalized to 2 nM, and then denatured using 0.1 N NaOH. Cluster amplification of denatured templates was performed in flow cells, in accordance with the manufacturer’s protocol (Illumina). Flow cells were paired-end sequenced on an Illumina HiSeq 2000 using HiSeq Sequencing kits. A base-calling pipeline (Sequencing Control Software (SCS), Illumina) was used to process the raw fluorescent images and the called sequences.

### Exome sequencing

WES was performed using SureSelect Human All Exon 44 Mb (Agilent), following the manufacturer’s standard protocol. Briefly, a paired-end DNA sequencing library was prepared through genomic DNA shearing, end-repair, A-tailing, PE adaptor ligation, and amplification. After hybridization of the library with bait sequences for 24 hours, the captured library was purified and amplified with an index barcode tag, and the library quality and quantity were determined. Sequencing of the exome library was carried out using the 100 bp paired-end mode of the HiSeq SBS kit, in accordance with the manufacturer’s manual.

### Read alignment and variation detection

Paired-end sequence reads were aligned to the hg19 human reference genome (NCBI build 37) with the Burrows-Wheeler Aligner (BWA) [69] (v0.5.9). Two mismatches were permitted in a 45 bp seed sequence. The rmdup command of SAMtools was used to remove PCR duplicates of sequence reads, which can be generated during the library construction process [70]. Aligned reads were realigned at putative indel positions with the Genome Analysis Toolkit (GATK) [71] IndelRealigner algorithm to enhance mapping quality. Base quality scores were recalibrated using the TableRecalibration algorithm of GATK.

### SNP and small insertion/deletion analysis and somatic mutation filtering

Putative SNVs were called and filtered using the UnifiedGenotyper and VariantFiltration commands in GATK. The options used for SNP calling were a read mapping depth of 5 to 200 times with a consensus quality of 20, and a prior likelihood for heterozygosity value of 0.001. To obtain small indels, the UnifiedGenotyper DINDEL mode of GATK was used with default values, including a window size of 300 bp. To identify somatic mutations in cancer genomes, mutations from cancer genomes were filtered using the mutations from blood genomes. The remaining mutations were filtered again using the mapping status of the blood genomes. At each remaining tumor mutation position, if the minimum mapping depth was at least 3 and the mutation ratio of the blood genome was at least 0.2, the tumor SNV was discarded. To remove false-positive reads caused by genomic duplications, the somatic mutations were called from uniquely mapped reads. Additionally, mutations located in duplicated sequences (≥90% identity) were filtered out if the mutations were not detected by both WGS and WES. The indels were called from reads aligned using the Smith-Waterman algorithm [72]. Two additional databases, dbSNP 131, and an internal Korean variation database that contains variations found in 20 healthy Koreans, were used to filter out additional SNVs. All somatic mutations altering amino acid sequences were checked by expert laboratory personnel using the tview command of SAMtools. The same method was applied to call SNVs and small indels from the previously reported exome data, except for the step filtering mutations located in duplicate sequences.

### Mutation rate calculation

For the mutation rate calculation, the number of mutations was compared with the total number of bases in sufficiently covered coding DNA sequence (CDS) regions. The mutations consisted of SNVs and small indels. The sufficiently covered CDS region was defined where its read mapping depth was at least five reads.

### Sanger sequencing

A total of 94 nsSNVs, indels, and SNVs in splice sites were verified by conventional Sanger sequencing using dye-terminator chemistry and analyzed with an automatic sequencer ABI 3730 (Applied Biosystems). The target regions were amplified by PCR followed by direct sequencing, or cloned into TA vectors. At least 20 TA vector clones were sequenced, because mutations in low purity samples are difficult to detect by Sanger sequencing. Details of the PCR and sequencing primers are given (see Additional file 1: Table S11).

### Annotation of variations

Predicted SNVs were compared with NCBI dbSNP (version 131) to annotate known SNP information. Each SNV was mapped on the University of California Santa Cruz (UCSC) gene table by genomic features such as coding region, untranslated region, and intron. Non-synonymous SNV information was extracted by comparing UCSC reference gene information. The KEGG pathway [73] was used to analyze altered protein sets. Information on cancer-related mutations was obtained from COSMIC (Catalogue of Somatic Mutations In Cancer) [74].

### Driver gene prediction

Driver gene scores were calculated using SNVs as described in a previous report [22], with an efficiently covered region with a normal sample mapping depth of 4 times or greater and a cancer sample mapping depth of 3 times or greater. In brief, the driver gene score was calculated by comparing the observed number of nsSNVs with the expected number. The expected number of nsSNVs was calculated from the background non-synonymous to synonymous SNV ratio, and the number of observed synonymous SNVs. The *P*-value for a driver gene score was calculated from the numbers of expected and observed nsSNVs, assuming that the numbers of nsSNVs had a Poisson distribution.

### Purity calculation

The purity of the cancer samples was determined by calculating the mapping depths ratio of the diploid and the haploid regions of the samples. Cancer samples usually have a mix of diploid and haploid regions, which are generated by heterozygous deletions. The first step is to calculate the ratio of the mapping depths from cancer and normal genomes by scanning the genomes with a window size of 50 kb. If a sample does not contain any tumor cells, there will always be only one peak in a histogram that shows the mapping depth ratio. If there are tumor cells, there will be two or more peaks (see Additional file 1: Figure S1). Secondly, the purity is calculated by the ratio distance(s) between the peaks using the equation shown below.

(1) $$\mathrm{Purity}\left(P\right)=\frac{2\left(R_{2n}‒R_{1n}\right)}{R_{2n}}$$

R_2n_ is the ratio of the diploid (2 N) region, and R_1n_ is the ratio of the haploid (1 N) region. When the purity was lower than about 0.5, the peaks were not distinct. To overcome this ambiguity, somatic deletion regions detected by BreakDancer were used as the 1 N depth regions [75]. The false-positive somatic deletion regions were filtered out using the deletion regions detected in the blood genomes. When the average depth ratio of the somatic deletion region was greater than that of the depth of the 2 N region, the deletion was regarded as a false positive.

### Identification of copy number variation regions

CNVs based on the differences in sequencing depths between normal and cancerous samples were detected using BIC-seq [76] v1.1.2 with λ = 100 and bin_size = 1000 bp. Regions with a log2 ratio smaller than −0.2 or larger than 0.2 were defined as deleted or duplicated regions, respectively. The CNV candidates were mapped to COSMIC [74] data to find cancer-associated genes. For previously reported array CGH data [32], +0.152173 and −0.135797 were applied as thresholds for gain and loss, respectively. Genes having its corresponding clones were used for CNV analysis.

### Identification of structural variants and gene fusions

SVs were scanned using BreakDancer [75] with score ≥80. A somatic SV was defined as an SV not found in blood samples. We obtained structural variation signals (SVSs), which are clusters composed of more than three uniquely and discordantly mapped read pairs from all SV regions. We used SVSs found only in tumor tissue samples for consecutive analysis. We considered two SVSs as equal, if the breakpoints of the two SVSs were 400 bp or closer to each other. SVSs located in intergenic and intron region were excluded. A gene was determined to have a breakage event when an SVS breakpoint occurred within the gene. Gene fusion was defined as a connection of two genes by a SVS. The final gene fusion candidates were selected when the number of supporting read pairs was above 10, and the only interchromosomal gene fusions were chosen.

### Structure prediction of CDH1

The mutated structure of CDH1 (E-cadherin) was predicted by homology modeling using mouse E-cadherin protein (PDBID:3Q2V) [77] as a template with MODELLER [78] v9.10.

### Genome-wide SNP analysis

SNP genotyping was performed using an Axiom genotyping solution with an Axiom Genome-Wide ASI 1 Array Plate and a reagent kit, in accordance with the manufacturer’s protocol (Affymetrix). Briefly, total genomic DNA (200 ng) was treated with 20 μl of denaturation buffer and 40 μl of neutralization buffer, followed by amplification for 23 hours using 320 μl of Axiom amplification mix. Amplified DNA was randomly digested into 25 to125 bp fragments with 57 μl of Axiom fragmentation mix at 37°C for 30 minutes, followed by DNA precipitation for DNA purification and recovery. DNA pellets were dried and resuspended in 80 μl of hybridization master mix, and 3 μl of suspended sample was used for sample qualification. A hybridization-ready sample was denaturated by PCR at 95°C for 20 minutes and 48°C for 3 minutes. The denatured DNA was transferred to a hybridization tray, and loaded onto a GeneTitan MC with an Axiom ASI array plate (Affytmerix). Hybridization continued on the GeneTitan for 24 hours, after which ligation, staining, and stabilization reagent trays were sequentially loaded onto the instrument. GeneTitan was controlled by an Affymetrix GeneChip Command Console GeneTitan Control (Affymetrix). The chip image was scanned with the GeneTitan, and the resulting data, a Image data (DAT) file, was automatically converted to a Cell Intensity data (CEL) file. The CEL intensity file was normalized, and genotype calling was performed using Genotyping Console 4.1 with Axiom GT1 algorithms, in accordance with the manufacturer’s manual. The cut-off value for data quality control was a DISHQC of 0.82 or greater for hybridization, and a call rate of 97% or greater.

### *MDM2* gene expression analysis by quantitative real-time PCR

*MDM2* mRNA expression was analyzed using a quantitative real-time PCR system, and the *MDM2* gene expression was normalized to *GAPDH*. Primer sequences for *MDM2* and *GAPDH* were as follows. *MDM2*-RT forward sequence was 5′-GGCCTGCTTTACATGTGCAA-3′, *MDM2*-RT reverse sequence was 5′-GCACAATCATTTGAATTGGTTGTC-3′, *GAPDH* forward sequence was 5′-TGCACCACCAACTGCTTA-3′, and *GAPDH* reverse sequence was 5′- GGATGCAGGGATGATGTTC-3′. Quantitative real-time PCR was performed with SYBR Green I PCR Master Mix (Qiagen) on a LightCycler 480 Real-Time PCR System (Roche). The experiments were performed in triplicate, and the PCR reaction was performed as follows: 5 minutes at 95°C for initial denaturation, then 45 cycles at 95°C for 10 seconds, 58°C for 10 seconds, and 72°C for 10 seconds, followed by melting curve analysis at 95°C for 5 seconds, 65°C for 1 minute, and cooling for 30 seconds at 40°C. For each reaction, 5 ng of cDNA, 500 nM primer (final concentration) and 5 μl of 2X SYBR Green I PCR Master Mix was used in a 10 μl reaction volume.

### Fusion gene analysis

Genomic rearrangement of the fusion gene was verified by PCR using a forward primer located in *TSC2* (5′-CTCAGGTTCCGAGCCTAACAG-3′) and a reverse primer in *RNF216* (5′-GCAAACATAGTGAGACCCCATCT-3′). The PCR reaction was performed as follows: 15 minutes at 94°C for initial denaturation, then 40 cycles at 94°C for 30 seconds, 60°C for 30 seconds, and 72°C for 1 minute, with 5 minutes at 72°C for post-extension. For each reaction, 30 ng/μl gDNA, 100 nM primer, and 0.5 U of Taq polymerase (Qiagen) were used in a 20 μl reaction. The expression of a fusion gene in one patient sample was analyzed by RT-PCR using a forward primer located in *TSC2* (5′-GAGCATGGCTCCTACAGGTACAC-3′) and a reverse primer in *RNF216* (5′-CTCTTCACAGGTGAGGCCATTAT-3′). The RT-PCR reaction was performed as follows: 5 minutes at 94°C for initial denaturation, then 40 cycles at 94°C for 30 seconds, 60°C for 30 seconds, and 72°C for 1 minute, with 5 minutes at 72°C for post-extension. For each reaction, 10 ng cDNA, 200 nM primer, and 0.5 U of Taq polymerase (Solgent, Korea) were used in each 20 μl reaction. The RT-PCR products were analyzed by Sanger sequencing using an automatic sequencer (ABI3700; Applied Biosystems) to verify their fusion at the sequence level.

### Data access

The data from this study have been submitted to NCBI Sequence Read Archive (SRA) [79] under accession number SRA057772 (WGS) and SRA057973 (WES).

## Abbreviations

3D: Three-dimensional; CGH: comparative genome hybridization; CNV: Copy number variation; EBV: Epstein-Barr virus; indel: Insertion or deletion; DGC: diffuse-type gastric cancer; Gb: Gigabase; HDGC: Hereditary diffuse-type gastric cancer; IGC: Intestinal-type gastric cancer; MSI: Microsatellite instability; mTOR: mammalian target of rapamycin; NF: nuclear factor; NGS: Next-generation sequencing; SNP: Single nucleotide polymorphism; nsSNV: Non-synonymous SNV; SNV: Single nucleotide variation; SV: Structural variation; SVS: Structural variation signal; Tb: Terabase; WES: Whole exome sequencing; WGS: Whole genome sequencing.

## Competing interests

The authors declare that they have no competing interests.

## Authors’ contributions

SHL and JSL: project leading and study supervision; YSL and JB: study concept and design; YSL, YSC, SL and JB: drafting of the manuscript; SL, YSC, SJ, HMK, BCG, HC and DH: analysis and interpretation of data; BCK and CHK: whole genome and exome sequencing data production; SHH, JAH and SyK: experimental data generation; GK and HLK: project design; GKL, YWK and IJC: clinical support and sample related works; and YK and KWK: sample preparation and analysis. All authors read and approved the final manuscript.

## Supplementary Material

Additional file 1 Figures S1 to S3 and Tables S1 to S20, in portable document format (pdf). Click here for file

Additional file 2 Variations in coding region (altering amino acids) and splice site. Click here for file

Additional file 3 Putative somatic gene breakages by structural variations. Click here for file

Additional file 4 Copy number variations. Click here for file

Additional file 5 Integrated alterations. Click here for file
