# Supplementary material for: Genomic profile analysis of diffuse-type gastric cancers
Source: Genome Biol. 2014 Apr 1;15(4):R55. doi: 10.1186/gb-2014-15-4-r55 (PMC4056347; doi:10.1186/gb-2014-15-4-r55)
Supplement: Additional file 1 — Figures S1 to S3 and Tables S1 to S20, in portable document format (pdf). [file gb-2014-15-4-r55-S1.pdf]

Supplementary Figures and Tables

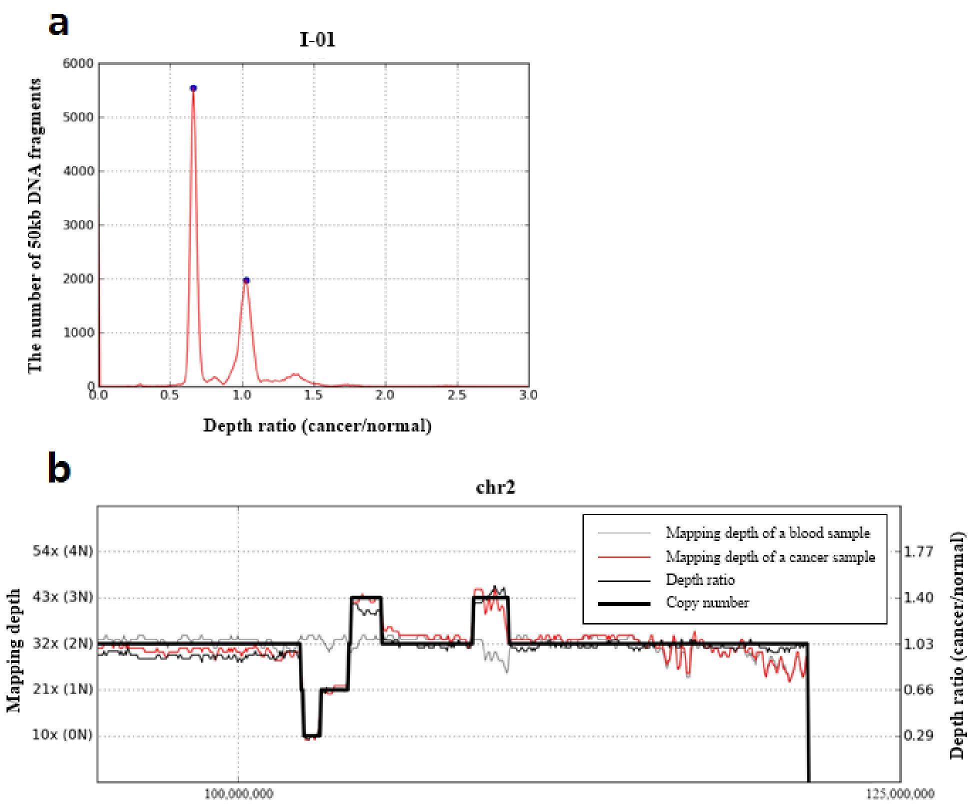

Figure S1. Depth ratio distributions (**a**) The depth ratio distribution of I-01. The two dots represent the peaks of the two most frequent depth ratios. (**b**) The mapping depth of the q-arm end region of I-01 chr2.

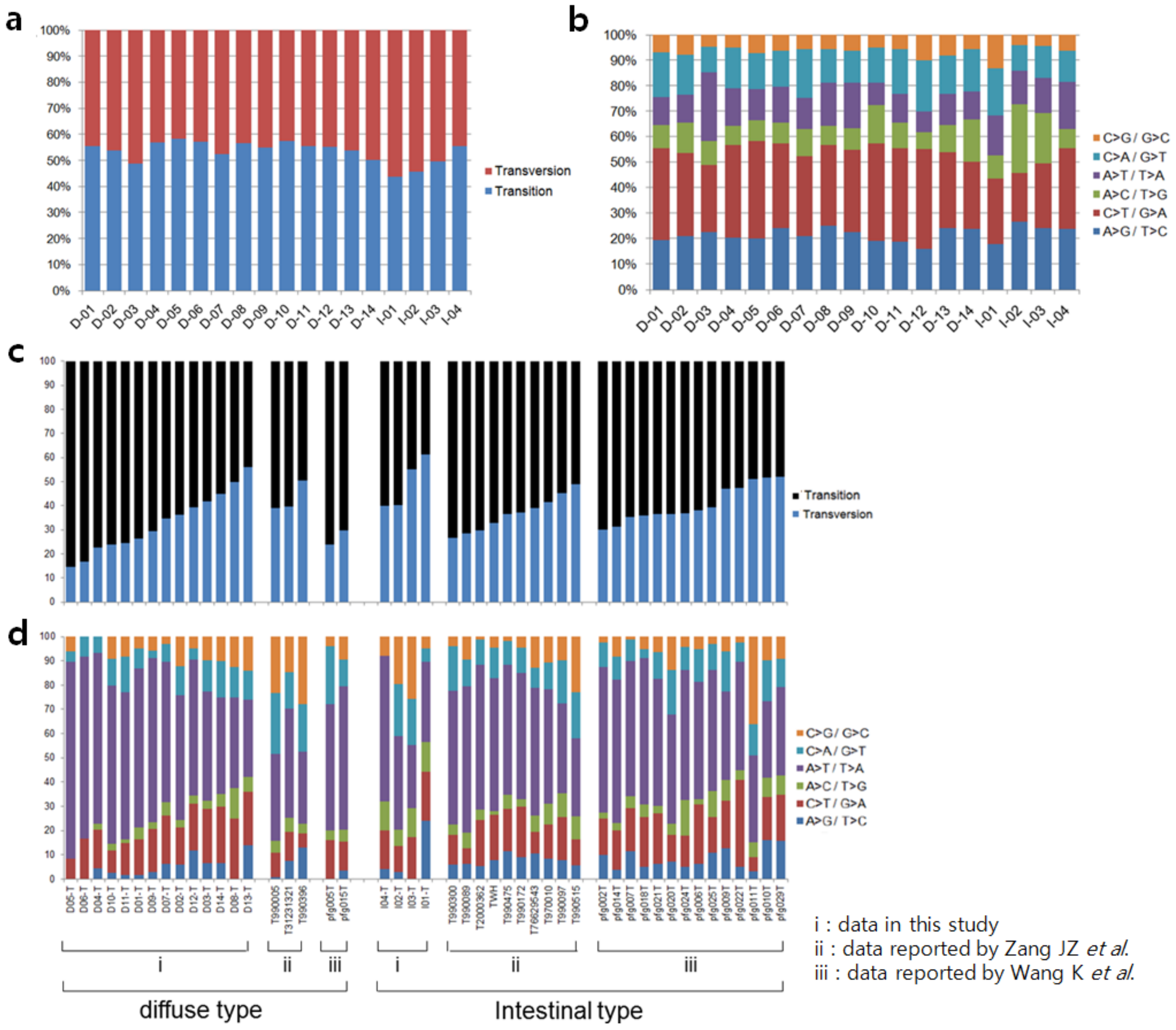

Figure S2. Somatic mutation profiles. (**a**) Transition and transversion ratio in whole genome. (**b**) Nucleotide substitution ratio in whole genome. (**c**) Transition and transversion ratio in exome region. (**d**) Nucleotide substitution ratio in exome region. The statistics in exome region were generated by combining data in this study together with previously reported exome data.

D-01T

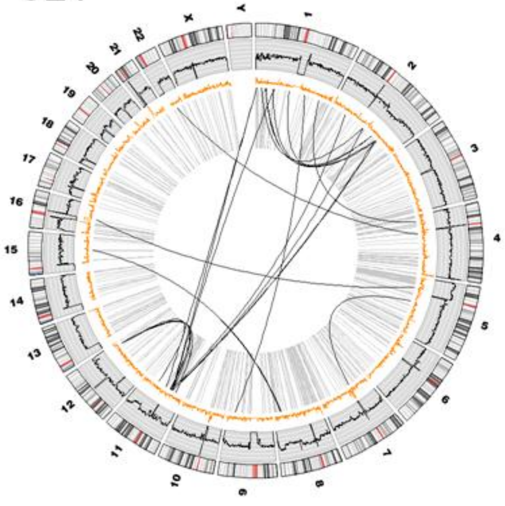

D-02T

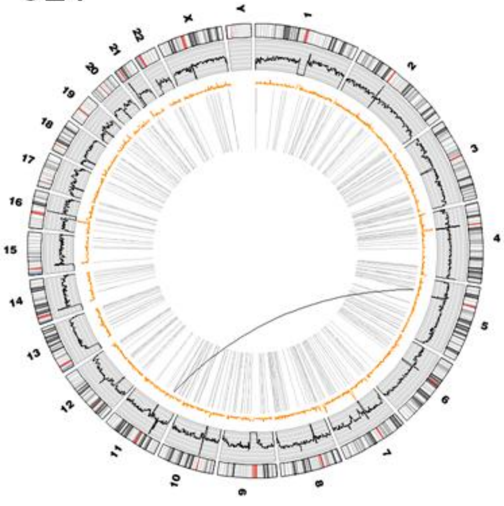

D-03T

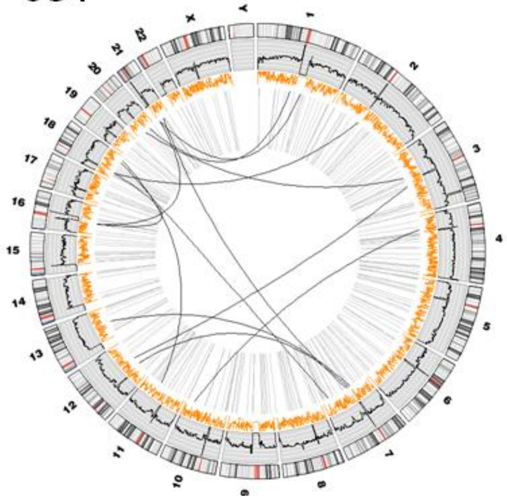

D-04T

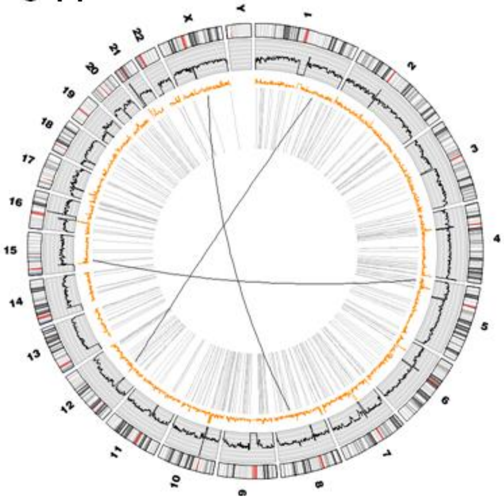

D-05T

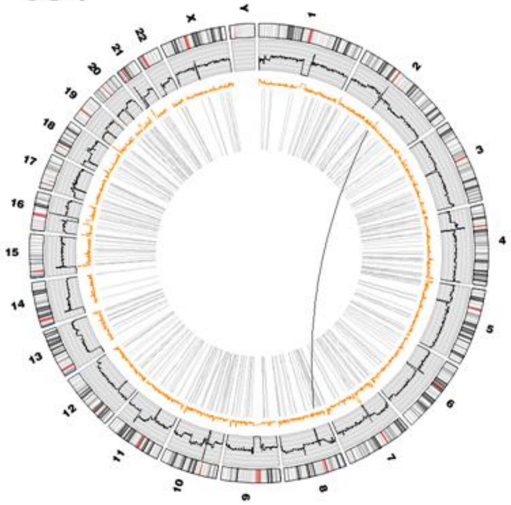

D-06T

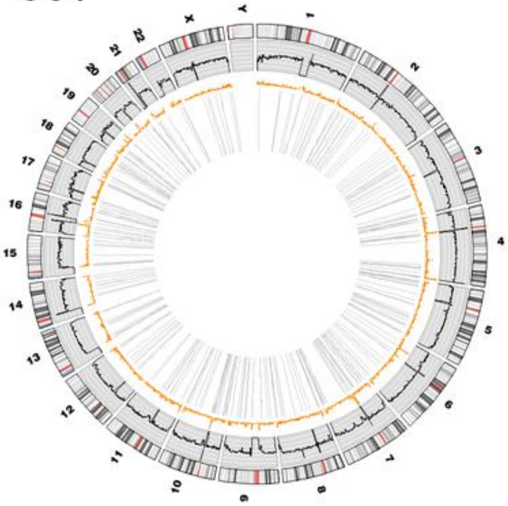

D-07T

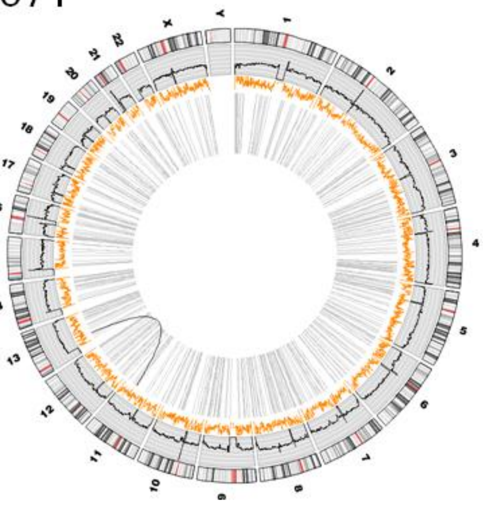

D-08T

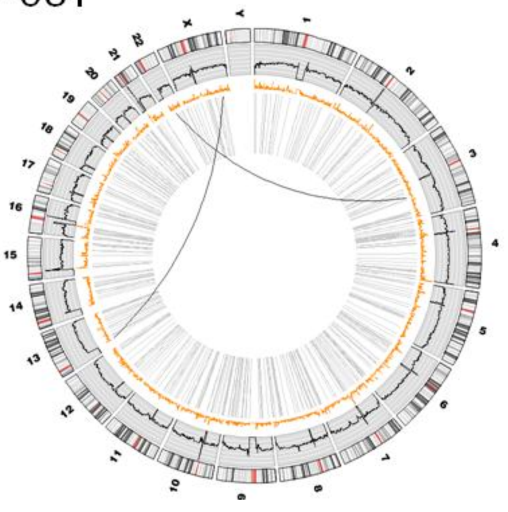

D-09T

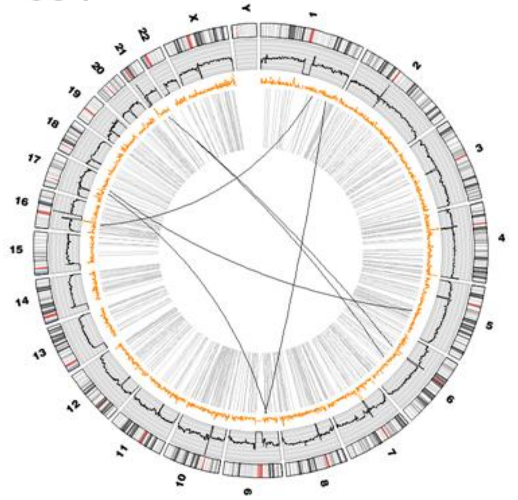

D-10T

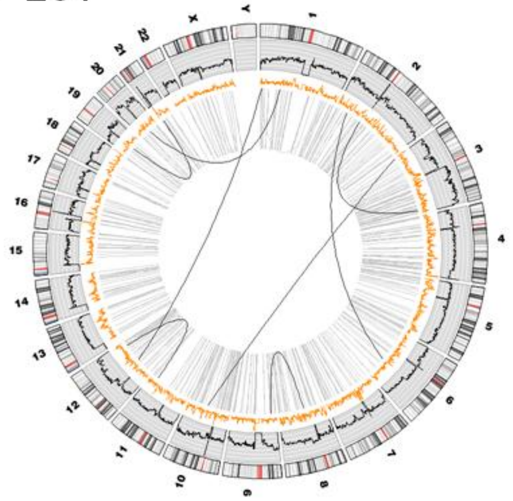

D-11T

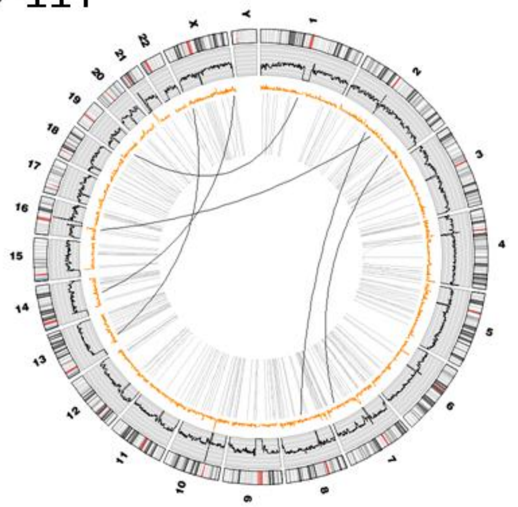

D-12T

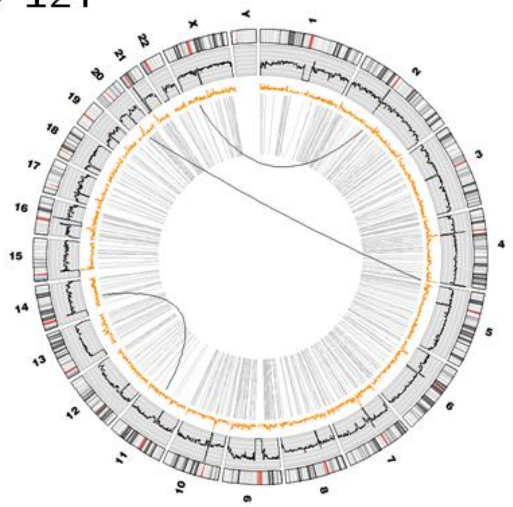

D-13T

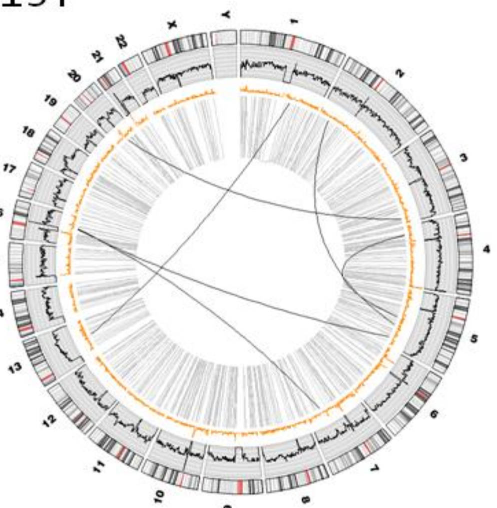

D-14T

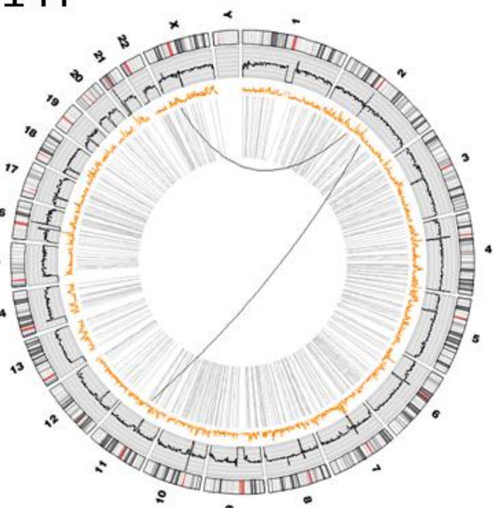

I-01T

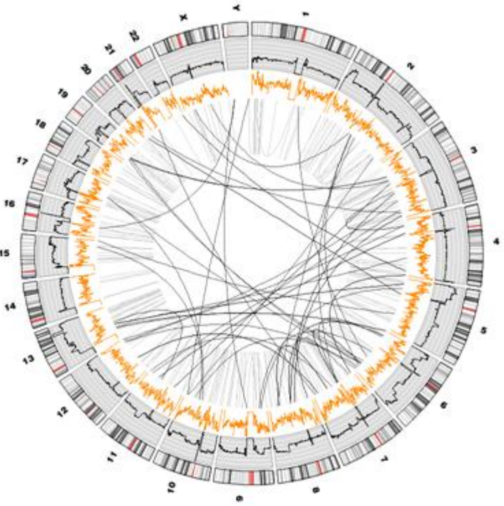

I-02T

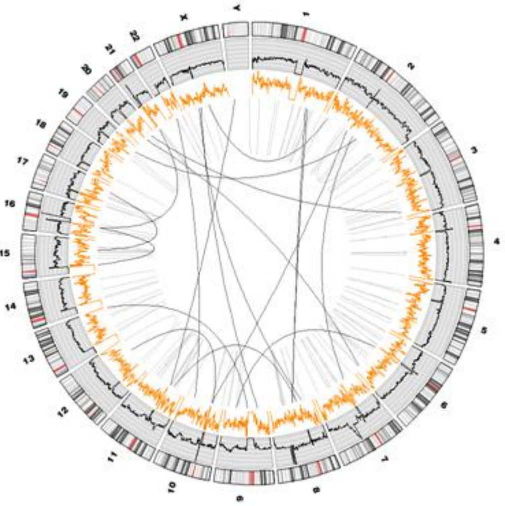

I-03T

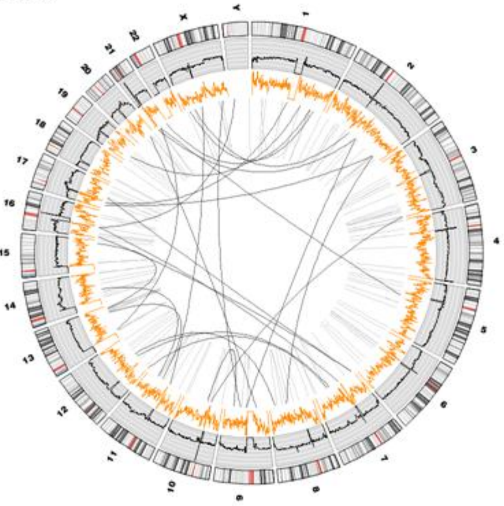

I-04T

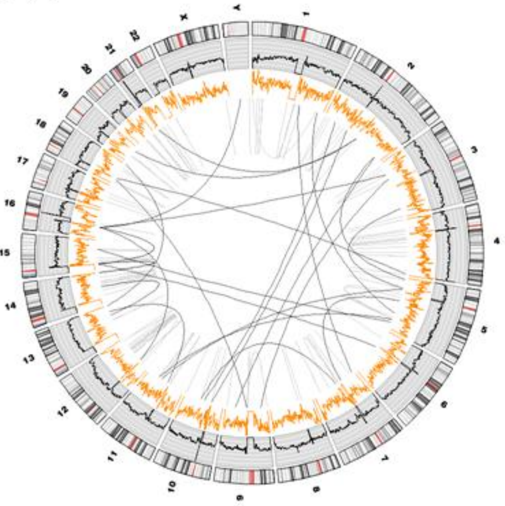

Figure S3. Structural variation of tumor genomes. From the outer side of each ring: chromosome ideograms, mapping depths of chromosome regions (black circles), somatic SNV density of chromosome regions (orange lines), and large translocations of intra- and inter-chromosomal rearrangements (gray lines in the center and black lines across the center, respectively).

Table S1. Clinico-pathological characteristics of gastric cancers from female patients younger than 50 years of age.

a. Diffuse type

Sample	Sex	Age	pTNMstage	Borrmann type	Location	Tumor size (Long Diameter)	H. pylori IgG
D-01	F	37	T3N2M0	3	cardia	6.00	not checked
D-02	F	34	T3N1M0	3	body	5.60	positive(17 UR/ml)
D-03	F	39	T3N2M0	3	antrum	9.00	positive(59 UR/ml)
D-04	F	37	T2bN2M0	3	high and mid body	8.00	positive(56 UR/ml)
D-05	F	37	T3N3M1(P1)	3	body	13.00	not checked
D-06	F	38	T3N2M0	3	body	13.00	not checked
D-07	F	38	T3N1M0	4	high and mid body	6.00	positive(>120 UR/ml)
D-08	F	34	T2bN1M0	3	cardia	5.00	positive(>120 UR/ml)
D-09	F	36	T3N0M0	3	body	5.50	positive(55 UR/ml)
D-10	F	42	T2N2M0	3	antrum	5.00	not checked
D-11	F	43	T3N2M0	2	body	5.50	negative (3 UR/ml)
D-12	F	43	T2bN0M0	2	cardia	6.00	positive(>120 UR/ml)
D-13	F	42	T3N1M1	3	body	5.00	positive(116 UR/ml)
D-14	F	41	T2bN1M0	3	antrum	3.20	positive(69 UR/ml)

b. Intestinal type

Sample	Sex	Age	pTNMstage	Borrmann type	Location	Tumor size (Long Diameter)	H. pylori IgG
I-01	F	42	T3N1M0	2	antrum	6.50	not checked
I-02	F	44	T3N3M0	3	antrum	5.00	positive(101 UR/ml)
I-03	F	27	T3N2M0	3	body	4.50	positive(67 UR/ml)
I-04	F	36	T3N0M0	3	body	5.50	not checked

c. MSI-Intestinal type

Sample	Sex	Age	pTNMstage	Borrmann type	Location	Tumor size (Long Diameter)	H. pylori IgG
M-01	F	47	T2bN0M0	2	antrum	10.50	positive(34 UR/ml)

Table S2. Sequencing statistics.

Cancer Type	Sample	total base (Gb)	mapped base (Gb)	sequencing depth (fold)	mapping depth (fold)	total reads	mapped reads
Diffuse type	D-01B	86.48	82.22	30.22	28.73	960,865,581	913,524,291 (95.07%)
	D-01T	97.53	93.66	34.09	32.73	1,083,718,112	1,040,634,002 (96.02%)
	D-02B	106.83	101.08	37.33	35.33	1,186,975,847	1,123,125,683 (94.62%)
	D-02T	94.84	91.02	33.15	31.81	1,053,828,338	1,011,362,470 (95.97%)
	D-03B	108.13	89.39	37.78	31.24	1,201,490,848	993,222,812 (82.67%)
	D-03T	111.65	90.83	39.01	31.73	1,105,499,128	899,326,770 (81.35%)
	D-04B	93.79	89.52	32.78	31.28	1,042,157,557	994,627,921 (95.44%)
	D-04T	87.84	85.13	30.7	29.75	976,017,163	945,939,153 (96.92%)
	D-05B	81.18	77.76	28.37	27.18	901,986,574	864,049,716 (95.79%)
	D-05T	94.31	90.76	32.96	31.72	1,047,836,756	1,008,397,034 (96.24%)
	D-06B	84.29	80.52	29.46	28.14	936,578,571	894,668,495 (95.53%)
	D-06T	104.06	99.73	36.37	34.86	1,156,184,318	1,108,141,790 (95.84%)
	D-07B	105.6	92.31	36.89	32.25	1,045,546,568	914,051,161 (87.42%)
	D-07T	82.67	79.77	28.89	27.88	918,606,800	886,296,876 (96.48%)
	D-08B	84.38	80.88	29.49	28.26	937,588,331	898,617,051 (95.84%)
	D-08T	86.31	82.84	30.16	28.95	958,946,893	920,459,735 (95.99%)
	D-09B	83.33	79.95	29.12	27.94	925,855,354	888,360,238 (95.95%)
	D-09T	85.48	82.58	29.88	28.86	949,832,205	917,550,733 (96.60%)
	D-10B	91.46	86.82	31.96	30.34	1,016,223,735	964,677,731 (94.93%)
	D-10T	88.22	84.93	30.83	29.68	980,254,769	943,661,255 (96.27%)
	D-11B	92.83	88.23	32.44	30.84	1,031,497,990	980,328,676 (95.04%)
	D-11T	84.13	81.2	29.4	28.38	934,818,450	902,230,978 (96.51%)
	D-12B	95.33	90.89	33.32	31.77	1,059,184,112	1,009,897,100 (95.35%)
	D-12T	99.37	95.29	34.73	33.3	1,104,126,335	1,058,774,473 (95.89%)
	D-13B	90.77	86.62	31.72	30.27	1,008,575,478	962,477,672 (95.43%)
	D-13T	97.48	87.99	34.06	30.75	1,083,142,004	977,640,141 (90.25%)
	D-14B	94.99	90.28	33.2	31.55	1,055,494,193	1,003,070,381 (95.03%)
	D-14T	96.64	93.05	33.77	32.52	1,073,743,176	1,033,856,700 (96.29%)
Intestinal type	I-01B	106.69	101.66	37.29	35.53	1,185,497,937	1,129,586,825 (95.28%)
	I-01T	84.92	81.14	29.68	28.36	943,519,000	901,546,422 (95.55%)
	I-02B	102.96	97.84	35.98	34.19	1,143,965,945	1,087,121,109 (95.03%)
	I-02T	79.24	75.88	27.69	26.52	880,405,197	843,122,159 (95.77%)
	I-03B	82.26	78.74	28.75	27.52	913,984,039	874,926,613(95.73%)
	I-03T	82.05	78.72	28.68	27.51	911,697,785	874,704,379(95.94%)
	I-04B	84.75	81.29	29.62	28.41	941,654,003	903,264,715 (95.92%)
	I-04T	81.92	78.98	28.63	27.6	910,240,469	877,526,355 (96.41%)
MSI-Intestinal type	M-01B	96.55	91.1	33.74	31.84	1,072,786,736	1,012,205,952 (94.35%)
	M-01T	93.61	90.17	32.71	31.51	1,040,083,970	1,001,929,798 (96.33%)

Table S3. Tumor sample purity.

a. Diffuse type

Sample	Purity	Sample	Purity
D-01T	0.51	D-08T	0.20
D-02T	0.33	D-09T	0.21
D-03T	0.41	D-10T	0.41
D-04T	0.38	D-11T	0.43
D-05T	0.39	D-12T	0.33
D-06T	0.18	D-13T	0.36
D-07T	0.33	D-14T	0.50

b. Intestinal type

Sample	Purity
I-01T	0.72
I-02T	0.49
I-03T	0.34
I-04T	0.17

c. MSI-Intestinal type

Sample	Purity
M-01T	0.58

Table S4. Concordance results between Axiom genotyping and SNVs.

Blood	Concordance	Tumor	Concordance
D-01B	99.83%	D-01T	99.77%
D-02B	99.84%	D-02T	99.79%
D-03B	99.85%	D-03T	99.83%
D-04B	99.87%	D-04T	99.31%
D-05B	99.87%	D-05T	99.72%
D-06B	99.84%	D-06T	90.85%
D-07B	99.84%	D-07T	99.82%
D-08B	99.84%	D-08T	99.85%
D-09B	99.85%	D-09T	99.81%
D-10B	99.84%	D-10T	97.94%
D-11B	99.84%	D-11T	99.50%
D-12B	96.21%	D-12T	99.81%
D-13B	99.84%	D-13T	99.84%
D-14B	99.83%	D-14T	99.83%
I-01B	99.85%	I-01T	91.96%
I-02B	99.86%	I-02T	99.16%
I-03B	99.84%	I-03T	99.82%
I-04B	99.86%	I-04T	99.83%

Table S5. SNVs in gastric cancer genomes.

Cancer Type	Sample		Total			Known (Valid in dbSNP131)							Known (not valid in dbSNP131)							Novel						
			Total	Known	Novel	PROMOTER	5UTR	CDS	SPLICING	3UTR	INTRON	nsSNP	PROMOTER	5UTR	CDS	SPLICING	3UTR	INTRON	nsSNP	PROMOTER	5UTR	CDS	SPLICING	3UTR	INTRON	nsSNP
Diffuse type	D-01	Blood	3,679,549	3,401,439	278,110	109,194	4,152	17,012	88	21,252	1,148,190	7,730	5,138	189	677	4	608	37,967	341	9,102	273	1153	18	1257	90,591	721
		Tumor	3,745,795	3,439,168	306,627	111,873	4,423	17,841	87	21,708	1,160,348	8,110	5,542	210	760	5	640	40,611	394	10,575	316	1,409	22	1,418	100,854	884
	D-02	Blood	3,739,807	3,436,242	303,565	112,772	4,641	18,137	85	22,078	1,169,935	8,225	5,458	263	798	5	677	41,115	424	10,684	337	1,405	31	1,488	100,533	889
		Tumor	3,700,718	3,412,268	288,450	110,085	4,401	17,389	77	21,646	1,160,345	7,938	5,102	248	710	5	660	39,494	365	9,368	299	1,228	20	1341	94,549	757
	D-03	Blood	3,745,398	3,448,085	297,313	113,463	4,765	18,559	85	21,622	1,166,602	8,375	5,608	244	787	2	708	41,839	431	10,118	318	1169	21	1,374	96,598	695
		Tumor	3,753,915	3,452,086	301,829	112,558	4,478	17,994	90	21,528	1,164,683	8,142	5,650	223	697	5	667	41,454	384	9,909	309	1,141	31	1,366	95,001	673
	D-04	Blood	3,756,367	3,460,809	295,558	114,673	5,088	19,083	92	22,085	1,168,179	8,633	5,761	263	822	5	624	41,795	451	10,036	372	1,259	20	1,390	95,775	753
		Tumor	3,758,802	3,458,082	300,720	113,849	4,953	18,883	92	22,013	1,166,340	8,567	5,581	243	795	4	631	41,698	446	9,976	370	1,261	25	1,414	97,157	785
	D-05	Blood	3,751,085	3,451,579	299,506	113,760	4,778	18,699	83	21,965	1,165,819	8,448	5,754	232	825	5	697	42,100	432	10,215	338	1,265	22	1,428	96,863	801
		Tumor	3,741,877	3,443,913	297,964	114,452	4,954	18,980	82	22,049	1,165,252	8,563	5,710	251	868	5	705	41,669	454	10,290	349	1,385	22	1,448	97,575	866
	D-06	Blood	3,773,769	3,471,003	302,766	115,186	5,216	18,885	91	22,162	1,176,701	8,567	5,954	270	805	5	703	42,846	418	10,482	394	1,289	22	1,441	99,897	765
		Tumor	3,760,408	3,462,774	297,634	115,165	5,113	18,782	89	22,123	1,175,207	8,479	5,893	264	808	7	672	42,052	419	10,360	383	1,317	20	1,400	98,355	773
	D-07	Blood	3,776,857	3,468,215	308,642	113,612	4,563	18,444	87	21,948	1,172,114	8,429	5,678	215	794	7	693	42,401	400	10,204	355	1,244	35	1,391	98,411	793
		Tumor	3,759,145	3,453,844	305,301	114,015	4,814	18,810	82	21,910	1,170,686	8,590	5,620	228	808	5	660	42,255	440	10,465	371	1,281	18	1,414	99,739	799
	D-08	Blood	3,748,017	3,449,068	298,949	112,849	4,880	18,771	94	21,885	1,167,518	8,591	5,647	237	857	4	675	41,953	456	10,413	364	1324	22	1,478	98,048	825
		Tumor	3,749,782	3,449,793	299,989	112,915	4,857	18,778	95	21,913	1,167,648	8,609	5,703	244	823	4	662	42,469	432	10,405	344	1,280	14	1,430	98,415	779
	D-09	Blood	3,743,186	3,444,461	298,725	113,595	4,867	18,519	87	22,065	1,169,422	8,420	5,560	208	783	2	704	41,536	417	10,061	368	1216	26	1,440	98,200	745
		Tumor	3,741,387	3,442,598	298,789	113,448	4,834	18,456	93	22,007	1,168,655	8,395	5,561	212	787	3	699	41,953	428	10,000	331	1172	17	1,448	97,828	707
	D-10	Blood	3,762,225	3,459,556	302,669	114,093	5,011	19,078	90	22,104	1,176,362	8,648	5,894	258	895	4	693	42,663	467	10,564	362	1,272	16	1,542	99,473	781
		Tumor	3,681,822	3,391,253	290,569	110,438	4,749	18,289	92	21,498	1,153,513	8,305	5,288	237	812	3	674	39,225	428	9,443	348	1,262	14	1,457	93,653	773
	D-11	Blood	3,710,964	3,426,281	284,683	113,509	5,149	19,101	86	22,048	1,163,479	8,624	5,594	276	829	5	721	40,074	436	10,137	390	1189	20	1400	92,734	720
		Tumor	3,696,226	3,412,734	283,492	111,529	4,861	18,433	79	21,825	1,157,727	8,351	5,396	254	776	4	656	39,199	413	9,537	368	1143	11	1375	91,107	695
	D-12	Blood	3,724,784	3,437,802	286,982	113,424	4,972	18,696	89	21,898	1,163,017	8,452	5,648	290	853	5	687	40,431	470	10,128	370	1,295	12	1401	93,012	809
		Tumor	3,725,741	3,432,749	292,992	112,953	4,916	18,500	89	21,771	1,161,246	8,388	5,586	285	821	6	658	40,225	439	10,049	352	1,327	22	1,455	94,567	834
	D-13	Blood	3,737,123	3,448,091	289,032	115,393	5,279	19,457	81	22,245	1,173,047	8,795	5,841	314	936	3	689	40,679	500	10,424	369	1,435	29	1334	95,941	875
		Tumor	3,717,936	3,437,169	280,767	113,325	4,964	18,789	81	22,005	1,168,450	8,498	5,525	271	792	5	666	39,532	409	9,615	296	1224	20	1306	92,046	742
	D-14	Blood	3,723,981	3,437,299	286,682	114,821	5,275	19,084	87	21,897	1,163,868	8,682	5,762	284	873	5	729	40,280	464	10,180	412	1,364	26	1345	93,846	846
		Tumor	3,742,843	3,446,322	296,521	114,010	5,071	18,647	87	21,849	1,165,407	8,502	5,767	264	871	4	733	40,569	468	10,241	383	1,365	24	1326	95,523	847
Intestinal type	I-01	Blood	3,736,388	3,444,644	291,744	114,964	5,031	18,872	88	22,095	1,166,896	8,582	5,776	269	813	6	695	40,736	429	10,351	364	1,287	14	1,430	95,290	779
		Tumor	3,540,324	3,247,849	292,475	106,166	4,595	17,196	85	20,517	1,097,292	7,859	5,290	262	747	7	640	38,242	396	9,795	357	1,273	19	1,404	94,509	783
	I-02	Blood	3,749,409	3,449,355	300,054	113,490	5,077	18,542	83	21,719	1,163,166	8,457	5,738	283	852	4	700	41,507	436	10,462	370	1214	25	1342	96,863	747
		Tumor	3,742,181	3,421,853	320,328	111,381	4,900	18,044	82	21,426	1,152,436	8,280	5,383	256	792	4	662	41,019	420	10,216	369	1,284	15	1,408	100,687	804
	I-03	Blood	3,766,762	3,465,515	301,247	114,833	4,955	18,689	87	22,027	1,171,168	8,480	5,844	238	847	6	695	42,477	459	10,266	360	1,200	25	1,407	98,003	758
		Tumor	3,762,412	3,459,480	302,932	114,992	5,067	18,777	92	22,078	1,169,501	8,495	5,806	254	836	6	697	41,988	466	10,225	379	1,234	20	1,401	97,640	769
	I-04	Blood	3,688,547	3,399,670	288,877	112,783	5,054	18,637	91	21,690	1,152,925	8,397	5,710	287	840	4	662	41,314	456	10,210	337	1,320	19	1340	93,873	799
		Tumor	3,744,648	3,447,776	296,872	113,109	4,946	18,508	86	21,821	1,164,674	8,378	5,695	253	801	5	711	42,055	432	10,032	309	1,271	19	1377	95,163	764
MSI-intestinal type	M-01	Blood	3,734,518	3,445,848	288,670	114,661	5,295	19,172	93	21,919	1,171,163	8,633	5,680	262	889	7	659	40,106	479	10,223	352	1,328	23	1,382	95,431	802
		Tumor	3,786,588	3,414,644	371,944	112,153	4,989	18,557	94	21,519	1,159,429	8,354	5,454	229	866	7	651	39,581	457	12,768	459	2,141	39	1,957	124,452	1,337

Table S6. Small indels in gastric cancer genomes.

Cancer type	Sample	Tissue	Total	PROMOTER	5UTR	CDS	SPLICING	3UTR	INTRON
Diffuse type	D-01	Blood	643,663	24,210	535	346	6	4,903	242,448
		Tumor	704,674	27,753	626	401	7	5,353	267,355
	D-02	Blood	706,185	28,351	629	373	3	5,460	269,990
		Tumor	673,305	25,336	592	318	3	5,139	254,504
	D-03	Blood	707,457	27,254	617	377	5	5,362	267,019
		Tumor	733.217	27,923	610	369	4	5,428	274,833
	D-04	Blood	693,592	27,546	684	409	4	5,378	261,387
		Tumor	698,650	26,768	660	388	5	5,366	262,196
	D-05	Blood	682,469	26,210	632	384	3	5,221	256,107
		Tumor	683,790	27,200	656	425	3	5,299	258,600
	D-06	Blood	696,127	27,140	649	393	3	5,248	263,242
		Tumor	714,221	28,022	655	401	6	5,389	271,097
	D-07	Blood	746,620	28,734	609	395	5	5,665	281,053
		Tumor	699,576	26,384	583	380	4	5,346	263,170
	D-08	Blood	681,734	25,823	602	376	5	5,218	256,529
		Tumor	704,496	26,828	623	381	5	5,393	266,116
	D-09	Blood	682,092	26,132	635	382	2	5,182	257,097
		Tumor	699,055	26,911	650	379	3	5,335	265,059
	D-10	Blood	613,344	24,099	591	360	3	4,854	232,094
		Tumor	649,740	24,440	597	359	5	5,013	245,233
	D-11	Blood	661,455	26,211	628	404	5	5,174	250,682
		Tumor	638,012	23,755	576	384	5	4,887	239,469
	D-12	Blood	679,860	26,638	659	382	6	5,307	257,796
		Tumor	683,625	26,220	641	384	5	5,266	258,133
	D-13	Blood	656,465	26,853	692	433	8	5,110	249,970
		Tumor	662,816	25,156	648	380	6	5,112	249,681
	D-14	Blood	648,771	26,165	699	395	6	5,033	245,532
		Tumor	683,598	26,781	649	376	5	5,217	258,156
Intestinal type	I-01	Blood	707,884	28,076	639	371	6	5,470	268,646
		Tumor	666,001	25,649	573	321	4	4,958	252,809
	I-02	Blood	719,737	28,511	669	388	7	5,425	272,223
		Tumor	678,271	25,709	594	353	5	5,033	253,796
	I-03	Blood	692,190	26,588	628	372	3	5,261	260,473
		Tumor	685,302	26,258	651	378	3	5,230	257,707
	I-04	Blood	673,267	26,534	663	394	5	5,193	253,630
		Tumor	683,877	25,800	637	374	4	5,143	256,426
MSI-Intestinal type	M-01	Blood	655,163	26,244	640	415	4	5,078	248,244
		Tumor	1,162,372	44,565	972	762	4	9,228	465,343

Table S7. Somatic mutations in gastric cancer genomes. Somatic mutations were mutations exclusively observed in cancer genomes.

Cancer type	Sample	PROMOTER	CDS			INTRON (SPLICING)	5'UTR	3'UTR	INTERGENIC	Total
			synSNV	nsSNV	indels					
Diffuse type	D-01T	343	14	46	3	3,491(1)	14	64	6,648	10,623
	D-02T	202	5	28	2	2,241(1)	13	39	4,207	6,737
	D-03T	829	15	41	0	6,580(4)	39	72	20,221	27,797
	D-04T	358	4	40	3	3,359(0)	17	38	6,414	10,233
	D-05T	312	8	40	5	2,899(0)	9	42	5,636	8,951
	D-06T	274	1	22	1	2,288(1)	9	35	4,501	7,131
	D-07T	565	17	77	1	5,255(2)	25	66	11,234	17,240
	D-08T	303	0	8	1	2,988(0)	11	30	5,632	8,973
	D-09T	377	3	30	0	3,707(1)	12	44	7,342	11,515
	D-10T	507	25	84	4	5,979(0)	24	92	12,761	19,476
	D-11T	253	13	47	1	2,739(2)	13	33	5,653	8,752
	D-12T	379	10	74	2	3,906(0)	20	79	7,942	12,412
	D-13T	239	7	42	1	2,525(1)	9	37	4,933	7,793
	D-14T	357	11	47	1	4,212(2)	18	55	10,002	14,703
Intestinal type	I-01T	943	48	112	4	9,499(3)	36	165	18,101	28,908
	I-02T	699	20	111	2	10,242(1)	33	122	25,703	36,932
	I-03T	416	6	52	1	4,600(0)	16	44	10,745	15,880
	I-04T	300	2	21	0	3,149(2)	8	38	5,912	9,430
MSI-Intestinal type	M-01T	3,331	260	725	455	33,731(88)	128	639	54,262	93,531

Table S8. Somatic mutation (SNVs and small indels) rate in CDS region.

a. Somatic mutation rate in 18 gastric cancers in this study

Sample Name	Sufficiently Covered CDS Region (bp)	# of Somatic Mutations (SNVs and small indels) in CDS	Somatic Mutation Rate (Mutations/Mb) in CDS
D-01T	32,243,491	62	1.92
D-02T	32,291,074	35	1.08
D-03T	32,304,182	53	1.64
D-04T	32,590,817	47	1.44
D-05T	32,650,298	53	1.62
D-06T	32,549,375	24	0.74
D-07T	32,381,825	94	2.9
D-08T	32,536,159	9	0.28
D-09T	32,509,756	33	1.02
D-10T	32,499,828	113	3.48
D-11T	32,562,459	61	1.87
D-12T	32,540,040	86	2.64
D-13T	32,615,890	49	1.5
D-14T	32,604,076	58	1.78
I-01T	32,570,715	164	5.04
I-02T	32,544,198	132	4.06
I-03T	32,521,490	59	1.81
I-04T	32,524,419	23	0.71
Mean of all samples : 1.97			Median of all samples : 1.71
Mean of diffuse-type samples : 1.71			Median of diffuse-type samples : 1.63
Mean of intestinal-type samples : 2.91			Median of intestinal-type samples : 2.94

b. Somatic mutation rate in 19 diffuse-type samples

Study	Sample	Sufficiently Covered CDS Region (bp)	# of Somatic Mutations (SNVs and small indels) in CDS	Somatic Mutation Rate (Mutations/Mb) in CDS
Data in this study	D-01T	32,243,491	62	1.92
	D-02T	32,291,074	35	1.08
	D-03T	32,304,182	53	1.64
	D-04T	32,590,817	47	1.44
	D-05T	32,650,298	53	1.62
	D-06T	32,549,375	24	0.74
	D-07T	32,381,825	94	2.90
	D-08T	32,536,159	9	0.28
	D-09T	32,509,756	33	1.02
	D-10T	32,499,828	113	3.48
	D-11T	32,562,459	61	1.87
	D-12T	32,540,040	86	2.64
	D-13T	32,615,890	49	1.50
	D-14T	32,604,076	58	1.78
Data reported by Wang K. *et al*.	T_pfg005	27,003,728	27	1.00
	T_pfg015	28,157,336	114	4.05
Data reported by Zang J.Z. *et al*.	T31231321	27,896,368	196	7.03
	T990005	28,849,574	113	3.92
	T990396	27,590,475	101	3.66
			Median :	1.78
			Mean :	2.29

c. Somatic mutation rate in 28 intestinal-type samples

Study	Sample	Sufficiently Covered CDS Region (bp)	# of Somatic Mutations (SNVs and small indels) in CDS	Somatic Mutation Rate (Mutations/Mb) in CDS
Data in this study	I-01T	32,570,715	164	5.04
	I-02T	32,544,198	132	4.06
	I-03T	32,521,490	59	1.81
	I-04T	32,524,419	23	0.71
Data reported by Wang K. *et al*.	T_pfg002	27,508,358	40	1.45
	T_pfg006	27,634,089	93	3.37
	T_pfg007	27,547,070	83	3.01
	T_pfg009	27,695,009	163	5.89
	T_pfg010	27,491,929	112	4.07
	T_pfg011	28,241,608	219	7.75
	T_pfg014	28,322,458	180	6.36
	T_pfg018	28,318,850	78	2.75
	T_pfg020	28,374,289	112	3.95
	T_pfg021	28,137,645	60	2.13
	T_pfg022	28,400,631	76	2.68
	T_pfg024	28,330,681	116	4.09
	T_pfg025	28,276,928	101	3.57
	T_pfg029	28,445,506	278	9.77
Data reported by Zang J.Z. *et al*.	T2000362	28,303,851	96	3.39
	T76629543	27,731,031	132	4.76
	T970010	28,224,015	134	4.75
	T990089	27,751,072	60	2.16
	T990097	28,830,854	40	1.39
	T990172	28,300,950	67	2.37
	T990300	27,388,652	50	1.83
	T990475	28,316,633	49	1.73
	T990515	28,223,216	196	6.94
	TWH	28,045,700	65	2.32
			Median :	3.39
			Mean :	3.71

Table S9. Cancer driver genes. Table for cancer driver genes in 14 diffuse-type samples was in main text Table 1.

a. Cancer driver genes in 4 intestinal-type samples (data in this study)

Gene Name	# of Samples	# of nsSNVs	# of SNVs in splicing site	# of indels	P-Value	Driver Gene Score
ACOT2	2	2	0	0	4.30E-07	5.00
ZNF138	1	1	0	0	4.88E-07	5.00
FOLH1	1	2	0	0	8.28E-07	4.95
LRRC8C	2	2	0	0	1.31E-06	4.87
TPO	1	2	0	0	2.66E-06	4.66
TMEM132D	1	2	0	0	4.37E-06	4.61
MMGT1	1	1	0	0	4.78E-06	4.61
NMU	1	1	0	0	7.05E-06	4.57

b. Cancer driver genes in 19 diffuse-type samples (data in this study and previously reported two exome data)

Gene Name	# of Samples	# of nsSNVs	# of SNVs in splicing site	# of indels	P-Value	Driver Gene Score
PIK3CA	6	6	0	0	5.75E-11	8.24
TP53	4	4	0	0	8.24E-10	7.38
CDH1	6	4	1	2(frameshift)	8.64E-08	5.54
SNRPN	2	2	0	0	3.88E-06	4.01
OR5C1	2	2	0	0	8.31E-06	3.77
CMKLR1	2	2	0	0	1.20E-05	3.70
GPR78	2	2	0	0	1.40E-05	3.69
CYP2A7	2	2	0	0	3.18E-05	3.40
GUCY1B3	2	2	0	0	5.41E-05	3.33
FAM71B	1	2	0	0	5.84E-05	3.33
SFTA3	1	1	0	0	5.85E-05	3.33
PAPOLB	2	2	0	0	5.86E-05	3.33
THEMIS	2	2	0	0	5.95E-05	3.33
LRFN5	2	2	0	0	7.85E-05	3.33
HIF3A	2	2	0	0	8.27E-05	3.33
SRXN1	1	1	0	0	8.31E-05	3.33
PAGE2	1	1	0	0	8.56E-05	3.33
C10orf90	2	2	0	0	8.60E-05	3.33
AKAP8	2	2	0	0	9.11E-05	3.33
NMB	1	1	0	0	9.32E-05	3.33

c. Cancer driver genes in 28 intestinal-type samples (data in this study and previously reported two exome data)

Gene Name	# of Samples	# of nsSNVs	# of SNVs in splicing site	# of indels	P-Value	Driver Gene Score
TP53	14	11	1	2(frameshift)	<7.37E-07	>4.09
PIK3CA	4	5	0	0	7.37E-07	4.09
ADAM18	3	4	0	0	2.40E-06	3.75
CTNNB1	3	4	0	0	4.35E-06	3.70
DBC1	3	4	0	0	4.44E-06	3.70
FMN2	4	5	0	0	1.29E-05	3.32
NOVA1	3	3	0	0	1.58E-05	3.30
DNAH7	5	7	0	1(frameshift)	2.42E-05	3.24
GREM2	2	2	0	0	2.57E-05	3.24
SPANXN2	2	2	0	0	2.58E-05	3.24
IL23A	2	2	0	0	3.17E-05	3.23
CYLC1	3	3	0	0	3.20E-05	3.23
SRSF2	2	2	0	0	4.26E-05	3.17
SLC39A12	2	3	0	0	4.28E-05	3.17
FOLH1	2	3	0	0	5.04E-05	3.13
ZNF138	1	1	0	0	6.16E-05	3.07
PDE4B	3	3	0	0	7.31E-05	3.05
LRRC8C	3	3	0	0	9.02E-05	3.05
CDH12	3	3	0	0	9.57E-05	3.05
OR5D13	2	2	0	0	9.60E-05	3.05

d. Diffuse-type specific cancer driver genes in 19 diffuse type samples (data in this study and previously reported two exome data)

Gene Name	# of Samples	# of nsSNVs	# of SNVs in splicing site	# of indels	P-Value	Driver Gene Score
CDH1	6	4	1	2(frameshift)	8.64E-08	5.54
SNRPN	2	2	0	0	3.88E-06	4.01
OR5C1	2	2	0	0	8.31E-06	3.77
CMKLR1	2	2	0	0	1.20E-05	3.70
GPR78	2	2	0	0	1.40E-05	3.69
CYP2A7	2	2	0	0	3.18E-05	3.40
GUCY1B3	2	2	0	0	5.41E-05	3.33
FAM71B	1	2	0	0	5.84E-05	3.33
SFTA3	1	1	0	0	5.85E-05	3.33
PAPOLB	2	2	0	0	5.86E-05	3.33
THEMIS	2	2	0	0	5.95E-05	3.33
LRFN5	2	2	0	0	7.85E-05	3.33
HIF3A	2	2	0	0	8.27E-05	3.33
SRXN1	1	1	0	0	8.31E-05	3.33
PAGE2	1	1	0	0	8.56E-05	3.33
C10orf90	2	2	0	0	8.60E-05	3.33
AKAP8	2	2	0	0	9.11E-05	3.33
NMB	1	1	0	0	9.32E-05	3.33

Table S10. Verified somatic nsSNV, small indels, and SNV in splicing sites.

Chr	Position	Gene	Allele change	Amino acid change	Sample Name	Result
chr3	137843404	*A4GNT*	-G	Q242fs	D-10T	positive
chr16	8866662	*ABAT*	G>A	R281Q	D-02T	positive
chrX	148037638	*AFF2*	T>C	I688T	D-14T	positive
chr13	42877846	*AKAP11*	T>C	V1655A	D-10T	positive
chr19	4207579	*ANKRD24*	G>A	A207T	D-07T	positive
chr1	3397035	*ARHGEF16*	C>T	R672W	D-10T	positive
chr14	104565259	*ASPG*	G>A	A195T	D-07T	positive
chrX	152830536	*ATP2B3*	G>A	R1106Q	D-10T	positive
chr16	28841304	*ATXN2L*	G>T	G320V	D-14T	positive
chr2	32712804	*BIRC6*	C>A	S2635Y	D-10T	positive
chr14	91662735	*C14orf159*	G>T	Q387H	D-14T	positive
chr1	170941024	*C1orf129*	G>A	D206N	D-07T	positive
chr4	15444383	*C1QTNF7*	C>A	T284K	D-14T	positive
chr1	209785156	*CAMK1G*	C>T	A312V	D-04T	positive
chr12	28459796	*CCDC91*	C>G	S130C	D-10T	positive
chr13	37012308	*CCNA1*	C>A	Q173K	D-07T	undefinable
chr16	68772218	*CDH1*	C>T	Q23*	D-12T	positive
chr16	68842472	*CDH1*	T>C	Splicing	D-03T	positive
chr16	68842726	*CDH1*	A>G	D221G	I-03T	positive
chr16	68844167	*CDH1*	T>G	V252G	D-09T	positive
chr16	68844179	*CDH1*	A>G	N256S	D-02T	positive
chr16	68844181	*CDH1*	G>A	D257N	D-05T	positive
chr16	68867236	*CDH1*	+T	S829fs	D-05T	positive
chr18	63525119	*CDH7*	A>T	T435S	D-13T	positive
chr7	92404109	*CDK6*	T>A	R90S	D-12T	positive
chr1	214816434	*CENPF*	G>A	E1585K	D-10T	positive
chr20	5904310	*CHGB*	G>T	S507I	D-10T	positive
chr13	53035988	*CKAP2*	C>T	R343*	D-10T	positive
chr12	108685988	*CMKLR1*	C>T	R251H	D-01T	positive
chr12	108686214	*CMKLR1*	C>T	V176I	D-10T	positive
chr2	99013441	*CNGA3*	G>A	R603Q	D-10T	positive
chr1	224377572	*DEGS1*	A>T	R126W	I-04T	positive
chr8	25237846	*DOCK5*	G>T	S1321I	D-14T	positive
chr6	46111322	*ENPP4*	G>A	R436H	D-10T	positive
chr10	126515341	*FAM175B*	A>G	R149G	D-09T	positive
chr9	128061234	*GAPVD1*	C>G	H12D	D-07T	positive
chr5	153174244	*GRIA1*	C>A	N778K	D-07T	negative
chr4	156715230	*GUCY1B3*	C>A	L240I	D-07T	positive
chrX	48675749	*HDAC6*	C>T	A603V	D-07T	positive
chr2	187541939	*ITGAV*	C>T	R987W	D-14T	positive
chr3	123953720	*KALRN*	C>T	R63C	D-03T	positive
chr1	152659422	*LCE2B*	-A	Q35fs	I-01T	positive
chr3	188327051	*LPP*	T>C	S178P	D-10T	positive
chr14	42360613	*LRFN5*	C>T	R516C	D-04T	positive
chr10	50121795	*LRRC18*	C>T	V136M	D-05T	positive
chr3	154866357	*MME*	G>T	E506*	D-06T	undefinable
chr4	103498166	*NFKB1*	G>T	A180S	D-10T	positive
chr1	159410278	*OR10J1*	C>T	R244W	D-02T	positive
chr14	20528604	*OR4L1*	C>A	T134K	D-12T	negative
chr11	55418468	*OR4S2*	C>A	S30Y	D-14T	positive
chr11	5969099	*OR56A3*	A>G	R175G	D-14T	positive
chr11	55563107	*OR5D14*	C>T	Q26*	D-10T	positive

chr5	78919211	*PAPD4*	C>T	H122Y	D-04T	positive
chr12	53848601	*PCBP2*	T>A	I6N	D-09T	undefinable
chr3	136002709	*PCCB*	C>T	Q192*	D-09T	positive
chr8	17847410	*PCM1*	A>G	S1485G	D-14T	positive
chr12	20787950	*PDE3A*	C>T	S654L	D-10T	positive
chr3	178936091	*PIK3CA*	G>A	E545K	D-04T	positive
chr3	178936091	*PIK3CA*	G>A	E545K	D-06T	positive
chr3	178936091	*PIK3CA*	G>A	E545K	D-12T	positive
chr3	178936094	*PIK3CA*	C>A	Q546K	D-11T	undefinable
chr3	178952085	*PIK3CA*	A>T	H1047L	D-02T	positive
chr12	130847595	*PIWIL1*	C>T	R701C	D-10T	positive
chr19	38865408	*PSMD8*	G>A	G56D	D-12T	undefinable
chr19	38867035	*PSMD8*	G>T	K159N	I-04T	negative
chr10	89685314	*PTEN*	-GTAA	Splicing	D-10T	positive
chr11	36594954	*RAG1*	C>T	R34W	D-07T	positive
chr15	59344506	*RNF111*	A>G	S295G	D-10T	positive
chr1	15987987	*RSC1A1*	G>A	V542I	D-10T	positive
chr3	38601696	*SCN5A*	-T	T1395fs	I-01T	positive
chrX	144904989	*SLITRK2*	A>C	Q349P	D-02T	positive
chr18	45368211	*SMAD2*	G>C	S434*	D-12T	positive
chr3	43344712	*SNRK*	G>A	R6Q	D-14T	positive
chr15	25223574	*SNRPN*	C>T	R236C	D-12T	positive
chr15	25223575	*SNRPN*	G>A	R236H	D-01T	positive
chr4	119951134	*SYNPO2*	C>T	R402W	D-10T	positive
chr17	7577547	*TP53*	C>G	G245A	D-13T	positive
chr17	7577574	*TP53*	T>G	Y236S	I-02T	positive
chr17	7578269	*TP53*	G>A	L194F	D-10T	positive
chr3	189526189	*TP63*	G>T	Q57H	D-13T	positive
chr2	1459951	*TPO*	A>G	H239R	I-02T	positive
chr2	1499949	*TPO*	G>C	W675S	D-02T	positive
chr2	1507810	*TPO*	C>T	A769V	D-01T	positive
chr2	1544478	*TPO*	G>T	A854S	I-02T	positive
chr8	15480637	*TUSC3*	C>T	R63C	D-04T	positive
chr2	128918093	*UGGT1*	G>T	L874F	D-09T	undefinable
chr8	117783987	*UTP23*	C>T	S219L	D-10T	positive
chr9	96021244	*WNK2*	C>T	T805M	D-07T	positive
chrX	64721879	*ZC3H12B*	G>A	R434H	D-02T	positive
chrX	64722658	*ZC3H12B*	C>T	R694C	D-07T	positive
chr7	64291841	*ZNF138*	G>A	V120I	I-01T	positive
chr19	35434621	*ZNF30*	C>A	Q251K	D-07T	positive
chr19	57089445	*ZNF470*	G>T	A550S	D-07T	positive
chr19	58806015	*ZNF8*	G>A	G281R	D-13T	undefinable

Table S11. Primers used for Sanger sequencing of nsSNVs, indels, SNVs in splicing site, CNVs, and the fusion gene.

Chr	Position	Gene	Sample Name	Forward primer sequence	Reverse primer sequence
chr3	137843404	*A4GNT*	D-10T	GAGATTTTCCACCAGTGTGTTGC	TTGATGACAAGGATGTTGAGGGT
chr16	8866662	*ABAT*	D-02T	TCACTGGTCTTAGGAAATGCACA	TGCAAGTCTGGAGATTGACAGAA
chrX	148037638	*AFF2*	D-14T	ATATTACCAGCAGCACTCCCAAA	CAGGGTCTCTTCCTGATCTGTGT
chr13	42877846	*AKAP11*	D-10T	CAGCCGCTATCAGAAATCTAGGA	ATGCAACAGGGAAAACTTTTGTT
chr19	4207579	*ANKRD24*	D-07T	GCACCCTTTGAAAGTCTGAGAAA	GGGCTACTGAGGAGATGAGAACA
chr1	3397035	*ARHGEF16*	D-10T	TGGTCAGTTCTAGGAGAAGCCTG	ACCACTTGCTGATTGGAGACAG
chr14	104565259	*ASPG*	D-07T	AGTACCTGCTCCCAGGAAGGTCT	AAGCATGGTGCTCTCAAACTCTG
chrX	152830536	*ATP2B3*	D-10T	GGGGACTGCTCTAGCTTTACTCC	CTCTACCCTCTTGGGGTGGAAGT
chr16	28841304	*ATXN2L*	D-14T	TGGTGATTGGACTTGTTGAAATG	ACAAGGCTTAAACCACAGCACTC
chr2	32712804	*BIRC6*	D-10T	GTCTCCCACTGGAACAGATGATT	TCATCCCTGACAGGATTAGAATGA
chr14	91662735	*C14orf159*	D-14T	GTGAATTTCCAAAAGCCAACACT	CAGGGACCTGTTACTTCCAGAGA
chr1	170941024	*C1orf129*	D-07T	AGCAAATGCATGTTCTCCTTTGT	TAACAGACGTAGAGCCTTCCCAG
chr4	15444383	*C1QTNF7*	D-14T	TGTGCTTTCCCAGGGATCTATTA	GAGTCTCTTTGGATCCCATTCCT
chr1	209785156	*CAMK1G*	D-04T	CCGTGTACCCTCTCTGAAATGAG	GGGTCTAGAGGTTTCTGAGGCTT
chr12	28459796	*CCDC91*	D-10T	GCAATCAACACACACTCATCTGG	TGCTTTAAACCTACAACCAAGGG
chr13	37012308	*CCNA1*	D-07T	TTTCCCCTATGCTGGTAGATTCA	TGGCATAAGAGTGCCATTCACTA
chr16	68772218	*CDH1*	D-12T	CTGTTGGTTTCGGTGAGCAG	CCACCACAGTGTTCAGTCGTAAA
chr16	68842472	*CDH1*	D-03T	CTGTACACTGCCCACAGAAGGCT	GATCCCAACACTGGGTCTTTTCC
chr16	68842726	*CDH1*	I-03T	GAGAAAGGGAAAAGACCCAGTGT	CTCCCATCACTTCTCCTTAGCAA
chr16	68844167	*CDH1*	D-09T	ATCAGAGCTCAAGTCACCCTCAC	CCAAGAAGTTCTGTCCGTAGGAA
chr16	68844179	*CDH1*	D-02T	ATCAGAGCTCAAGTCACCCTCAC	CCAAGAAGTTCTGTCCGTAGGAA
chr16	68844181	*CDH1*	D-05T	ATCAGAGCTCAAGTCACCCTCAC	CCAAGAAGTTCTGTCCGTAGGAA
chr16	68867236	*CDH1*	D-05T	CTGGGTGCATTGTCGTACCTTAC	CTCTCTCGAGTCCCCTAGTCGTC
chr18	63525119	*CDH7*	D-13T	TAAACCAGGCATGCATTTATGTG	CTCCACTCACTGTCCTCCATCTT
chr7	92404109	*CDK6*	D-12T	CTCCAGGCTCTGGAACTTTATCC	GATGTTCTCATGCTTAACCGCAC
chr1	214816434	*CENPF*	D-10T	CTCTTGTGTGCCTGACAGCTCTA	CTGTTGCCACTGTTCATTTTCTG
chr20	5904310	*CHGB*	D-10T	GTGTCCAAGAAAACCAGATGGAC	TTCTTTCAAAATGGCTGCTCTTC
chr13	53035988	*CKAP2*	D-10T	GAAAGGGCCTCATGAAAAAGAAC	TGCCTGCATATGACAGAAGAGAA
chr12	108685988	*CMKLR1*	D-01T	GGAGCTCTAGGAGGTTGAGTGTG	GGTCCTGGCTTTCTTCTTGAGTT
chr12	108686214	*CMKLR1*	D-10T	TAGCAAGCTGTGATGATGAGGAC	TTCTCATCCACAACATGTTCACC
chr2	99013441	*CNGA3*	D-10T	GCTACTCAGACCTGTTCTGCCTC	TGCATTTTCACTGTTGTTTGTCC
chr1	224377572	*DEGS1*	I-04T	TATTCATGAGATTGCCCACAATG	TCTGAAAGCGGTACAGAAGAACC
chr8	25237846	*DOCK5*	D-14T	TTTTGCCACTGAATCAATGACTG	CACTCCACTTCCAGAGATGTCCT
chr6	46111322	*ENPP4*	D-10T	GTGGTGCATTAATCTCCCAGAAG	CCGAAACAAAACACAAGGAAAAC
chr10	126515341	*FAM175B*	D-09T	CAGCAGATGTCCTACAGAGAGCA	ACCACACCCAGCTAATTTTTGAA
chr9	128061234	*GAPVD1*	D-07T	AAGTGCTGGGATTATAGGCATGA	AAATTGATTCTCTGTTGCTTCGC
chr5	153174244	*GRIA1*	D-07T	TGCTGAGTGTTCTCCACCTGTTA	ATTGGGAGAACAGTTGCTGGTTA
chr4	156715230	*GUCY1B3*	D-07T	ACGCATCAGCCCATATACATTCT	AGGGAAAGTTGTGGTATGCTCAA
chrX	48675749	*HDAC6*	D-07T	AGACCATCCTGGATAACATGGTG	AGCCACAGAGTTGAAAAAGCAAA
chr2	187541939	*ITGAV*	D-14T	GGGAAGGAAGAAAGGGACTGTAA	TGCCAATTAGCACTATCAGCAGT
chr3	123953720	*KALRN*	D-03T	CCCAGCACTCAGACAGATTTCTT	GTAACAATGGGAATGAGGCAGAC
chr1	152659422	*LCE2B*	I-01T	CACCTAAGTGTCCCCCTAAATGC	GAAAGGGTTAAGCATCAGGCTGT
chr3	188327051	*LPP*	D-10T	CGTTGGCAGTAATTTTTGCTTTC	AAAGGTAGGCCTTGAAGAAGTGG
chr14	42360613	*LRFN5*	D-04T	ACAAATTCCATTGTCCCTCAGAA	GACCTTGTGTTGCCCATTATTGT
chr10	50121795	*LRRC18*	D-05T	TCCGCTTTATGTTGAGCTTTTTC	ACATAGACAAGCTGCCTGAGTCC
chr3	154866357	*MME*	D-06T	GTGATTTGGCTGGAATACTGACC	AAGGAGGGAAAGACCTGCTTCTA
chr4	103498166	*NFKB1*	D-10T	TAAAGCATTGAGGGCCTGTGTAT	GTAGAGACTGGAAGGGATGCAGA
chr1	159410278	*OR10J1*	D-02T	TTATCAGTGTGCTGGTGCTTGTT	CTGCCTAGTGTGGATGTTGACAG
chr14	20528604	*OR4L1*	D-12T	TCAACCCTTCATTCTCCCTTGTA	TCACAAGGGGAAGATCACAAAAT
chr11	55418468	*OR4S2*	D-14T	TCCTAAGAACTTGACCCATTCCA	CCACATAACGATCATAGGCCATT
chr11	5969099	*OR56A3*	D-14T	GGCCTATGATCGTTATGTAGCCA	GGATGAGGTCAGATCCTAGCAGA
chr11	55563107	*OR5D14*	D-10T	TATTTTGCTTTGGCAGGAACAAT	CTTGGGAGTGACAATGGAAGAGT
chr5	78919211	*PAPD4*	D-04T	GCTTGTTTTCAGGAGATTAAGCG	CTTATCTTTGGCCTCAGGCAGT

chr12	53848601	*PCBP2*	D-09T	TCCTCCTCTGATTTTGGTCATGT	GTCAAGTCTGTCCATTTTCCCAC
chr3	136002709	*PCCB*	D-09T	TGCCTCAAACATCTCTGTAACCA	GCTACAGATTTCATGCCAAAACC
chr8	17847410	*PCM1*	D-14T	TCTTGTTTGGACAGAATACTGAGGA	ACTTGTTTCCTTATCAACGCCAA
chr12	20787950	*PDE3A*	D-10T	TGATGTTCTCCACCCTATGATCC	TCAAAGATTCATTTCTGAGCCAA
chr3	178936091	*PIK3CA*	D-04T	TCCAGAGGGGAAAAATATGACAA	GCTGAGATCAGCCAAATTCAGTT
chr3	178936091	*PIK3CA*	D-06T	TCCAGAGGGGAAAAATATGACAA	GCTGAGATCAGCCAAATTCAGTT
chr3	178936091	*PIK3CA*	D-12T	TCCAGAGGGGAAAAATATGACAA	GCTGAGATCAGCCAAATTCAGTT
chr3	178936094	*PIK3CA*	D-11T	TCCAGAGGGGAAAAATATGACAA	GCTGAGATCAGCCAAATTCAGTT
chr3	178952085	*PIK3CA*	D-02T	TGACATTTGAGCAAAGACCTGAA	GTGTGGAATCCAGAGTGAGCTTT
chr12	130847595	*PIWIL1*	D-10T	TGGGGTTGCCATTCTACTCTCTA	GGTTCTCCCTCATCCTTTCATCT
chr19	38865408	*PSMD8*	D-12T	GTCACCATCTTGAGTGACGACAG	CTAAGATTGGGGCTTTTACGGTT
chr19	38867035	*PSMD8*	I-04T	GAAGAGACCTCAGTCACCTCCTG	TAGTCAAGCCAAGTCTCAGGACC
chr10	89685314	*PTEN*	D-10T	TGGTGGCTTTTTGTTTGTTTGTT	TGTGCCAACAATGTTTTACCTCA
chr11	36594954	*RAG1*	D-07T	GACTCAGTTCTGCCCCAGATG	CTTTTAACAATGGCTGAGTTGGG
chr15	59344506	*RNF111*	D-10T	CCATAGAAGAGGAAGTAAACATGCAA	TCTCACCGATAGCTTTCTCCAAC
chr1	15987987	*RSC1A1*	D-10T	GCACTCAATCAGACTTCTGAGCA	AAAACAAGAAGTGCAAGGTCTGC
chr3	38601696	*SCN5A*	I-01T	ACCACAGACCATCCCCATACTCT	ACAAGAGCCAGTGTGAGTCCTTG
chrX	144904989	*SLITRK2*	D-02T	CGAGTGACTGTGTCAAAGGACAG	GCAGTCCATCAAACATAGAAGGG
chr18	45368211	*SMAD2*	D-12T	GGACCACACACAATGCTATGACA	TAAACATAGCCATTTTCCCTGCC
chr3	43344712	*SNRK*	D-14T	TCCATGACGACATTGAAAATGAA	CAATAACTTCATAAAGGCGGACG
chr15	25223574	*SNRPN*	D-12T	AGGCCCCTGAATATGTGTATCCT	CAACTGCTTAATAGGCATCACCC
chr15	25223575	*SNRPN*	D-01T	AGGCCCCTGAATATGTGTATCCT	CAACTGCTTAATAGGCATCACCC
chr4	119951134	*SYNPO2*	D-10T	CATTTCTGAGTTGCTGTGGTGTC	TTGCACCAAGAAATGCTACTTCA
chr17	7577547	*TP53*	D-13T	GAAAAGAAAACTGAGTGGGAGCA	GGGCCTGTGTTATCTCCTAGGTT
chr17	7577574	*TP53*	I-02T	GACCTGGAGTCTTCCAGTGTGAT	TGAAACCCCGTCTCTACTGAAAA
chr17	7578269	*TP53*	D-10T	GAGGTCAAATAAGCAGCAGGAGA	CCATCTACAAGCAGTCACAGCAC
chr3	189526189	*TP63*	D-13T	CATGGACCAGCAGATTCAGAAC	TTCCCACTAACTGGCACTTTCTC
chr2	1459951	*TPO*	I-02T	GTCCTCCTATGTCCTGACCAATG	TTTCCAGTGAGATGCACCAGATA
chr2	1499949	*TPO*	D-02T	TGTCTCCTTCTCTGGAGGTTTTG	AATCAGCTCCTGGGGAAGATAAG
chr2	1507810	*TPO*	D-01T	CTGTCTGCTCCTCATCACCTTTT	CAAATGGACGGACAGAGAGAGAG
chr2	1544478	*TPO*	I-02T	ACTAAATCCACACTGCCCATCTC	TCACATTCTGGCTCACTGTCATT
chr8	15480637	*TUSC3*	D-04T	GCATTCTTGGATTGCTGACTTCT	ATCATGGAATAGTTTCGAGGTGG
chr2	128918093	*UGGT1*	D-09T	GGGAGACTGGAGAATTGACACAT	TGCAAAGACTGTTCACGGATCTA
chr8	117783987	*UTP23*	D-10T	AAGAAGCCTGGAGTTCCTCTCAT	CATTCTCCTTCTGCATTCTGCTT
chr9	96021244	*WNK2*	D-07T	GCCTGCCAACTTTCCTTTTCTAT	ATCACGGCTGGAGAGAAATACTG
chrX	64721879	*ZC3H12B*	D-02T	ACAGAGGGTTCCATCACTGAGAG	GGAACACTTAGGGCAGTCACAAG
chrX	64722658	*ZC3H12B*	D-07T	ACAAACAGTCAGTCCCCCACTTA	GGGATAGGAGTGGTAGCCATAGG
chr7	64291841	*ZNF138*	I-01T	GGTATTTTGCTATGCCATCTTGC	CCTTGGTGTCCCTTACACTCATC
chr19	35434621	*ZNF30*	D-07T	TGGTGAGAAGCCACTCAAATGTA	GCATTCGTAAGGTTTTTCACTGG
chr19	57089445	*ZNF470*	D-07T	TATCTGTGAGAAAGCCTTCAGCC	CCACTGTGGAGTCTCTGATGTTG
chr19	58806015	*ZNF8*	D-13T	ATTACGGAACTCACAAAAAGCCA	TCCTGACACTCATAGGGCTTCTC
CNV		*MDM2*	D-01T, D-02T	GGCCTGCTTTACATGTGCAA	GCACAATCATTTGAATTGGTTGT
Fusion gene		*TSC2-RNF216*	I-03T	gDNA: GCAAACATAGTGAGACCCCATCT	CTCAGGTTCCGAGCCTAACAG
				RNA: GAGCATGGCTCCTACAGGTACAC	CTCTTCACAGGTGAGGCCATTAT

Table S12. KEGG pathways enriched in mutated genes in 14 diffuse- and 4 intestinal type samples.

a. KEGG pathway enriched in mutated genes in 14 diffuse-types

KEGG Pathway	KEGG Pathway Name	P-value
path:hsa05222	Small cell lung cancer	4.70E-05
path:hsa00460	Cyanoamino acid metabolism	6.90E-05
path:hsa04020	Calcium signaling pathway	7.00E-05
path:hsa04930	Type II diabetes mellitus	3.09E-04
path:hsa00500	Starch and sucrose metabolism	3.27E-04
path:hsa05213	Endometrial cancer	9.03E-04
path:hsa00524	Butirosin and neomycin biosynthesis	1.69E-03
path:hsa04512	ECM-receptor interaction	1.86E-03
path:hsa05200	Pathways in cancer	2.00E-03
path:hsa04530	Tight junction	2.08E-03
path:hsa04510	Focal adhesion	3.20E-03
path:hsa00630	Glyoxylate and dicarboxylate metabolism	3.44E-03
path:hsa04974	Protein digestion and absorption	4.00E-03
path:hsa05218	Melanoma	4.15E-03
path:hsa04910	Insulin signaling pathway	4.40E-03
path:hsa05217	Basal cell carcinoma	4.74E-03
path:hsa05214	Glioma	4.74E-03
path:hsa05146	Amoebiasis	5.80E-03
path:hsa04720	Long-term potentiation	6.10E-03
path:hsa04740	Olfactory transduction	6.66E-03
path:hsa04730	Long-term depression	6.88E-03
path:hsa05014	Amyotrophic lateral sclerosis (ALS)	7.72E-03
path:hsa05130	Pathogenic Escherichia coli infection	8.00E-03
path:hsa04914	Progesterone-mediated oocyte maturation	9.14E-03
path:hsa00232	Caffeine metabolism	9.60E-03
path:hsa04970	Salivary secretion	1.10E-02
path:hsa00640	Propanoate metabolism	1.14E-02
path:hsa04973	Carbohydrate digestion and absorption	1.14E-02
path:hsa00360	Phenylalanine metabolism	1.15E-02
path:hsa04012	ErbB signaling pathway	1.20E-02
path:hsa05219	Bladder cancer	1.34E-02
path:hsa04310	Wnt signaling pathway	1.37E-02
path:hsa05210	Colorectal cancer	1.45E-02
path:hsa04320	Dorso-ventral axis formation	1.45E-02
path:hsa00010	Glycolysis / Gluconeogenesis	1.50E-02
path:hsa04960	Aldosterone-regulated sodium reabsorption	1.55E-02
path:hsa00400	Phenylalanine, tyrosine and tryptophan biosynthesis	1.56E-02
path:hsa04120	Ubiquitin mediated proteolysis	1.73E-02
path:hsa00280	Valine, leucine and isoleucine degradation	1.87E-02
path:hsa04742	Taste transduction	2.05E-02
path:hsa05412	Arrhythmogenic right ventricular cardiomyopathy (ARVC)	2.09E-02
path:hsa05220	Chronic myeloid leukemia	2.26E-02
path:hsa05212	Pancreatic cancer	2.26E-02
path:hsa04540	Gap junction	2.26E-02
path:hsa00430	Taurine and hypotaurine metabolism	2.27E-02
path:hsa05414	Dilated cardiomyopathy	2.29E-02
path:hsa05223	Non-small cell lung cancer	2.30E-02
path:hsa04110	Cell cycle	2.38E-02
path:hsa04514	Cell adhesion molecules (CAMs)	2.44E-02
path:hsa04722	Neurotrophin signaling pathway	2.44E-02
path:hsa04670	Leukocyte transendothelial migration	2.59E-02
path:hsa00350	Tyrosine metabolism	2.64E-02
path:hsa04966	Collecting duct acid secretion	3.06E-02
path:hsa00062	Fatty acid elongation in mitochondria	3.10E-02
path:hsa00051	Fructose and mannose metabolism	4.11E-02
path:hsa00982	Drug metabolism - cytochrome P450	4.11E-02
path:hsa05410	Hypertrophic cardiomyopathy (HCM)	4.30E-02
path:hsa00410	beta-Alanine metabolism	4.69E-02

b. KEGG pathway enriched in mutated genes in 4 intestinal-types

KEGG Pathway	KEGG Pathway Name	P-value
path:hsa00524	Butirosin and neomycin biosynthesis	4.25E-04
path:hsa04740	Olfactory transduction	5.27E-04
path:hsa04540	Gap junction	7.78E-04
path:hsa05218	Melanoma	1.00E-03
path:hsa04730	Long-term depression	1.59E-03
path:hsa00830	Retinol metabolism	4.46E-03
path:hsa04662	B cell receptor signaling pathway	4.86E-03
path:hsa05219	Bladder cancer	5.53E-03
path:hsa00010	Glycolysis / Gluconeogenesis	6.13E-03
path:hsa00051	Fructose and mannose metabolism	6.56E-03
path:hsa04914	Progesterone-mediated oocyte maturation	6.60E-03
path:hsa04144	Endocytosis	7.02E-03
path:hsa05214	Glioma	8.16E-03
path:hsa05213	Endometrial cancer	8.16E-03
path:hsa04976	Bile secretion	8.17E-03
path:hsa05223	Non-small cell lung cancer	8.93E-03
path:hsa04973	Carbohydrate digestion and absorption	9.98E-03
path:hsa04610	Complement and coagulation cascades	1.28E-02
path:hsa00740	Riboflavin metabolism	1.39E-02
path:hsa04971	Gastric acid secretion	1.68E-02
path:hsa05160	Hepatitis C	1.79E-02
path:hsa05210	Colorectal cancer	1.80E-02
path:hsa05216	Thyroid cancer	1.94E-02
path:hsa00350	Tyrosine metabolism	1.94E-02
path:hsa00512	Mucin type O-Glycan biosynthesis	2.07E-02
path:hsa04910	Insulin signaling pathway	2.20E-02
path:hsa05020	Prion diseases	2.34E-02
path:hsa00600	Sphingolipid metabolism	2.34E-02
path:hsa04360	Axon guidance	2.67E-02
path:hsa04070	Phosphatidylinositol signaling system	2.77E-02
path:hsa04964	Proximal tubule bicarbonate reclamation	2.86E-02
path:hsa00565	Ether lipid metabolism	2.86E-02
path:hsa04270	Vascular smooth muscle contraction	2.93E-02
path:hsa03022	Basal transcription factors	3.24E-02
path:hsa05110	Vibrio cholerae infection	3.24E-02
path:hsa04630	Jak-STAT signaling pathway	3.34E-02
path:hsa04060	Cytokine-cytokine receptor interaction	3.36E-02
path:hsa04115	p53 signaling pathway	3.49E-02
path:hsa04970	Salivary secretion	3.49E-02
path:hsa04340	Hedgehog signaling pathway	3.50E-02
path:hsa04012	ErbB signaling pathway	3.68E-02
path:hsa00760	Nicotinate and nicotinamide metabolism	3.75E-02
path:hsa04370	VEGF signaling pathway	3.76E-02
path:hsa01040	Biosynthesis of unsaturated fatty acids	4.22E-02
path:hsa04310	Wnt signaling pathway	4.27E-02
path:hsa04916	Melanogenesis	4.48E-02
path:hsa05217	Basal cell carcinoma	4.60E-02
path:hsa00030	Pentose phosphate pathway	4.72E-02
path:hsa04612	Antigen processing and presentation	4.91E-02

Table S13. KEGG pathways enriched in mutated genes in combined 19 diffuse- and 28 intestinal-type samples.

a. KEGG pathway enriched in mutated genes in 19 diffuse-types

KEGG Pathway	KEGG Pathway Name	P-value
path:hsa05222	Small cell lung cancer	1.00E-06
path:hsa04510	Focal adhesion	2.00E-06
path:hsa05200	Pathways in cancer	6.00E-06
path:hsa05218	Melanoma	1.40E-05
path:hsa04020	Calcium signaling pathway	5.50E-05
path:hsa05213	Endometrial cancer	1.12E-04
path:hsa04512	ECM-receptor interaction	1.53E-04
path:hsa05146	Amoebiasis	2.37E-04
path:hsa00460	Cyanoamino acid metabolism	2.55E-04
path:hsa04012	ErbB signaling pathway	3.59E-04
path:hsa05214	Glioma	5.81E-04
path:hsa05223	Non-small cell lung cancer	7.08E-04
path:hsa05210	Colorectal cancer	8.21E-04
path:hsa04930	Type II diabetes mellitus	8.28E-04
path:hsa04730	Long-term depression	1.03E-03
path:hsa05219	Bladder cancer	1.26E-03
path:hsa05220	Chronic myeloid leukemia	1.80E-03
path:hsa05212	Pancreatic cancer	1.80E-03
path:hsa00640	Propanoate metabolism	1.96E-03
path:hsa00630	Glyoxylate and dicarboxylate metabolism	2.45E-03
path:hsa04914	Progesterone-mediated oocyte maturation	3.14E-03
path:hsa00500	Starch and sucrose metabolism	3.18E-03
path:hsa04910	Insulin signaling pathway	3.39E-03
path:hsa00524	Butirosin and neomycin biosynthesis	4.03E-03
path:hsa04810	Regulation of actin cytoskeleton	4.37E-03
path:hsa05211	Renal cell carcinoma	4.87E-03
path:hsa04740	Olfactory transduction	5.57E-03
path:hsa04144	Endocytosis	5.57E-03
path:hsa04540	Gap junction	6.30E-03
path:hsa04320	Dorso-ventral axis formation	7.24E-03
path:hsa04514	Cell adhesion molecules (CAMs)	7.58E-03
path:hsa05100	Bacterial invasion of epithelial cells	8.64E-03
path:hsa00280	Valine, leucine and isoleucine degradation	8.93E-03
path:hsa05217	Basal cell carcinoma	1.03E-02
path:hsa04973	Carbohydrate digestion and absorption	1.08E-02
path:hsa05215	Prostate cancer	1.10E-02
path:hsa00300	Lysine biosynthesis	1.16E-02
path:hsa04120	Ubiquitin mediated proteolysis	1.16E-02
path:hsa05130	Pathogenic Escherichia coli infection	1.19E-02
path:hsa04530	Tight junction	1.22E-02
path:hsa04970	Salivary secretion	1.25E-02
path:hsa04720	Long-term potentiation	1.35E-02
path:hsa04960	Aldosterone-regulated sodium reabsorption	1.57E-02
path:hsa05014	Amyotrophic lateral sclerosis (ALS)	1.74E-02
path:hsa04722	Neurotrophin signaling pathway	1.93E-02
path:hsa04966	Collecting duct acid secretion	1.97E-02
path:hsa00232	Caffeine metabolism	2.22E-02
path:hsa05142	Chagas disease (American trypanosomiasis)	2.28E-02
path:hsa00010	Glycolysis / Gluconeogenesis	2.42E-02
path:hsa05145	Toxoplasmosis	2.68E-02
path:hsa05410	Hypertrophic cardiomyopathy (HCM)	2.68E-02
path:hsa05412	Arrhythmogenic right ventricular cardiomyopathy (ARVC)	2.69E-02
path:hsa00532	Glycosaminoglycan biosynthesis - chondroitin sulfate	2.90E-02
path:hsa00051	Fructose and mannose metabolism	2.90E-02
path:hsa04145	Phagosome	3.03E-02
path:hsa04660	T cell receptor signaling pathway	3.12E-02
path:hsa04310	Wnt signaling pathway	3.25E-02
path:hsa04724	Glutamatergic synapse	3.37E-02
path:hsa00071	Fatty acid metabolism	3.38E-02
path:hsa04670	Leukocyte transendothelial migration	3.44E-02
path:hsa00400	Phenylalanine, tyrosine and tryptophan biosynthesis	3.54E-02
path:hsa00360	Phenylalanine metabolism	3.64E-02
path:hsa04974	Protein digestion and absorption	3.70E-02
path:hsa04270	Vascular smooth muscle contraction	3.89E-02
path:hsa05414	Dilated cardiomyopathy	4.17E-02
path:hsa04070	Phosphatidylinositol signaling system	4.92E-02

b. KEGG pathway enriched in mutated genes in 28 intestinal-types

KEGG Pathway	KEGG Pathway Name	P-value
path:hsa04971	Gastric acid secretion	<1.00E-06
path:hsa04540	Gap junction	<1.00E-06
path:hsa04730	Long-term depression	1.00E-06
path:hsa04720	Long-term potentiation	4.90E-05
path:hsa04512	ECM-receptor interaction	6.60E-05
path:hsa05146	Amoebiasis	8.50E-05
path:hsa04912	GnRH signaling pathway	8.60E-05
path:hsa04510	Focal adhesion	1.04E-04
path:hsa05412	Arrhythmogenic right ventricular cardiomyopathy (ARVC)	1.07E-04
path:hsa04514	Cell adhesion molecules (CAMs)	1.66E-04
path:hsa04972	Pancreatic secretion	2.17E-04
path:hsa04910	Insulin signaling pathway	2.88E-04
path:hsa04270	Vascular smooth muscle contraction	3.18E-04
path:hsa04020	Calcium signaling pathway	3.25E-04
path:hsa04320	Dorso-ventral axis formation	3.43E-04
path:hsa05414	Dilated cardiomyopathy	3.81E-04
path:hsa04916	Melanogenesis	5.34E-04
path:hsa04360	Axon guidance	6.01E-04
path:hsa05200	Pathways in cancer	6.08E-04
path:hsa04070	Phosphatidylinositol signaling system	9.33E-04
path:hsa04914	Progesterone-mediated oocyte maturation	1.13E-03
path:hsa05215	Prostate cancer	1.16E-03
path:hsa05218	Melanoma	1.30E-03
path:hsa05210	Colorectal cancer	1.57E-03
path:hsa05213	Endometrial cancer	1.67E-03
path:hsa05410	Hypertrophic cardiomyopathy (HCM)	2.40E-03
path:hsa04964	Proximal tubule bicarbonate reclamation	2.59E-03
path:hsa04350	TGF-beta signaling pathway	2.91E-03
path:hsa04724	Glutamatergic synapse	3.77E-03
path:hsa04970	Salivary secretion	4.54E-03
path:hsa04962	Vasopressin-regulated water reabsorption	4.55E-03
path:hsa04960	Aldosterone-regulated sodium reabsorption	5.77E-03
path:hsa04144	Endocytosis	7.09E-03
path:hsa00562	Inositol phosphate metabolism	7.53E-03
path:hsa05320	Autoimmune thyroid disease	1.28E-02
path:hsa00640	Propanoate metabolism	1.28E-02
path:hsa04973	Carbohydrate digestion and absorption	1.28E-02
path:hsa04940	Type I diabetes mellitus	1.28E-02
path:hsa05145	Toxoplasmosis	1.28E-02
path:hsa04976	Bile secretion	1.32E-02
path:hsa04012	ErbB signaling pathway	1.32E-02
path:hsa04974	Protein digestion and absorption	1.48E-02
path:hsa00524	Butirosin and neomycin biosynthesis	1.49E-02
path:hsa04810	Regulation of actin cytoskeleton	1.50E-02
path:hsa04520	Adherens junction	1.52E-02
path:hsa05216	Thyroid cancer	1.57E-02
path:hsa05330	Allograft rejection	2.16E-02
path:hsa04740	Olfactory transduction	2.16E-02
path:hsa04330	Notch signaling pathway	2.60E-02
path:hsa05212	Pancreatic cancer	2.66E-02
path:hsa00410	beta-Alanine metabolism	2.77E-02
path:hsa04310	Wnt signaling pathway	2.99E-02
path:hsa04742	Taste transduction	3.20E-02
path:hsa00310	Lysine degradation	3.55E-02
path:hsa04530	Tight junction	4.05E-02
path:hsa00300	Lysine biosynthesis	4.10E-02
path:hsa05217	Basal cell carcinoma	4.13E-02
path:hsa05214	Glioma	4.13E-02
path:hsa05222	Small cell lung cancer	4.24E-02
path:hsa05221	Acute myeloid leukemia	4.70E-02
path:hsa04966	Collecting duct acid secretion	4.72E-02
path:hsa05110	Vibrio cholerae infection	4.78E-02

Table S14. Structural variation counts. Structural variations were detected using the BreakDancer program with a score >=30. Insertions were not found in D-03T and D-07B samples, because their insert size was about 300bp. DEL is large scale deletion, INS is large scale insertion, INV is inversion, ITX is intra-chromosomal translocation, and CTX is inter-chromosomal translocation.

a. Diffuse type

Sample	SV Type	all of Normal	all of Tumor	somatic of Tumor >= 80
D-01T	DEL	6171	6607	400
	INS	2585	2406	166
	INV	235	357	38
	ITX	274	440	44
	CTX	375	680	27
D-02T	DEL	7551	5916	180
	INS	3173	3063	224
	INV	272	374	32
	ITX	308	285	23
	CTX	564	461	1
D-03T	DEL	8054	3465	196
	INS	4543	0	0
	INV	283	406	51
	ITX	250	6814	152
	CTX	401	596	15
D-04T	DEL	6662	6467	75
	INS	3614	4202	318
	INV	266	589	4
	ITX	313	298	21
	CTX	438	396	3
D-05T	DEL	8156	5953	299
	INS	4870	2689	131
	INV	380	484	30
	ITX	301	439	42
	CTX	428	359	1
D-06T	DEL	5799	5297	196
	INS	1965	2841	103
	INV	398	487	15
	ITX	400	550	28
	CTX	570	458	0
D-07T	DEL	3601	6109	210
	INS	0	3937	387
	INV	350	913	15
	ITX	3610	300	36
	CTX	535	609	1
D-08T	DEL	5937	4648	215
	INS	4713	3163	260
	INV	325	752	23
	ITX	293	312	37
	CTX	427	1123	2
D-09T	DEL	6970	7412	367
	INS	4599	2819	242
	INV	538	1318	45
	ITX	272	307	34
	CTX	404	703	6

b. Intestinal type

Sample	SV Type	all of Normal	all of Tumor	somatic of Tumor >= 80
I-01T	DEL	5648	7536	569
	INS	4202	4403	443
	INV	277	283	29
	ITX	288	293	35
	CTX	375	540	61
I-02T	DEL	6681	8142	387
	INS	3347	3183	229
	INV	259	315	33
	ITX	273	295	32
	CTX	414	591	7
I-03T	DEL	8146	5860	119
	INS	4650	3722	165
	INV	425	690	40
	ITX	296	365	57
	CTX	403	473	16
I-04T	DEL	6436	6159	56
	INS	4144	4722	301
	INV	489	512	32
	ITX	300	308	27
	CTX	412	524	13

D-10T	DEL	7361	6841	280
	INS	3283	3100	278
	INV	253	334	18
	ITX	249	262	34
	CTX	334	390	6
D-11T	DEL	6356	6307	158
	INS	4263	2830	114
	INV	243	390	18
	ITX	279	284	20
	CTX	380	372	8
D-12T	DEL	6643	6338	352
	INS	3660	2815	268
	INV	253	271	16
	ITX	291	285	40
	CTX	445	404	3
D-13T	DEL	6536	6375	426
	INS	2853	2675	236
	INV	241	396	53
	ITX	289	327	61
	CTX	320	451	6
D-14T	DEL	6986	6373	345
	INS	2747	3652	233
	INV	257	296	19
	ITX	303	325	41
	CTX	351	397	2

Table S15. Somatic gene breakages.

a. Diffuse type

Sample	breakages	genes
D-01T	250	213
D-02T	113	101
D-03T	331	209
D-04T	45	38
D-05T	57	53
D-06T	54	49
D-07T	213	194
D-08T	613	570
D-09T	334	310
D-10T	92	62
D-11T	57	40
D-12T	55	49
D-13T	242	195
D-14T	389	216

b. Intestinal type

Sample	breakages	genes
I-01T	292	198
I-02T	202	158
I-03T	158	114
I-04T	98	89

Table S16. Somatic fusion gene candidates list.

Chromosome 1	Gene 1	strand 1	Chromosome 2	Gene 2	strand 2	# of supporting read pairs
chr1	*CHD5*	-	chr2	*METTL8*	-	16
chr1	*DDAH1*	-	chr8	*CSMD3*	-	11
chr1	*PRDM16*	+	chr11	*STX5*	-	23
chr1	*TRIM62*	-	chr2	*PDE1A*	-	11
chr2	*CYP1B1-AS1*	+	chr6	*PREP*	-	12
chr2	*MYO7B*	+	chr8	*PPP2R2A*	+	15
chr2	*OSBPL6*	+	chr11	*LRRC4C*	-	18
chr3	*SLC9A10*	-	chr8	*KCNB2*	+	29
chr5	*PDE4D*	-	chr11	*TRIM66*	-	12
chr5	*PRKAA1*	-	chr11	*DLG2*	-	10
chr5	*RAD1*	-	chr9	*DENND4C*	+	21
chr6	*C6orf114*	-	chr20	*CDH4*	+	10
chr6	*GFOD1*	-	chr20	*CDH4*	+	10
chr6	*VTA1*	+	chr17	*MPP2*	-	16
chr7	*RNF216*	-	chr16	*TSC2*	+	11
chr7	*RNF216*	-	chr17	*ERBB2*	+	10
chr8	*KLHL38*	-	chr15	*CCNB2*	+	21
chr11	*KIAA1731*	+	chr12	*SPPL3*	-	14
chr11	*PTPRJ*	+	chr12	*UNC119B*	+	12

Table S17. Diffuse-type enriched copy number variations.

Gene Name	All samples (data in this study and array CGH data by Tsukamoto *et al*.) Intestinal Type 28 Samples			Intestinal Type 20 Samples			Diffuse-type enriched CNVs	
	Loss	Neutral	Gain	Loss	Neutral	Gain	CNV Type	P-value
PTPRB	3	15	10	6	14	0	Gain	2.01E-03
PTPRR	4	15	9	6	14	0	Gain	4.12E-03
IFNG	3	18	7	6	14	0	Gain	1.61E-02
IL22	3	18	7	6	14	0	Gain	1.61E-02
IL26	3	18	7	6	14	0	Gain	1.61E-02
NUP107	2	19	7	7	13	0	Gain	1.61E-02
PPM1H	5	16	7	8	12	0	Gain	1.61E-02
SLC35E3	2	19	7	7	13	0	Gain	1.61E-02
GLIPR1	6	16	6	7	13	0	Gain	3.07E-02
KRR1	6	16	6	7	13	0	Gain	3.07E-02
LEMD3	5	17	6	6	14	0	Gain	3.07E-02
MSRB3	5	17	6	6	14	0	Gain	3.07E-02
TRHDE	4	18	6	10	10	0	Gain	3.07E-02
NLRP13	3	13	12	6	11	3	Gain	3.90E-02
NLRP4	3	13	12	6	11	3	Gain	3.90E-02
NLRP5	3	13	12	6	11	3	Gain	3.90E-02
NLRP8	3	13	12	6	11	3	Gain	3.90E-02
ZNF135	3	13	12	5	12	3	Gain	3.90E-02
ZNF274	3	13	12	5	12	3	Gain	3.90E-02
ZNF329	3	13	12	5	12	3	Gain	3.90E-02
ZSCAN1	3	13	12	5	12	3	Gain	3.90E-02
ZSCAN18	3	13	12	5	12	3	Gain	3.90E-02
CPM	2	18	8	6	13	1	Gain	4.12E-02
MDM2	2	18	8	6	13	1	Gain	4.12E-02
CACNG6	3	15	10	7	11	2	Gain	4.24E-02
CACNG7	3	15	10	7	11	2	Gain	4.24E-02
CACNG8	3	15	10	7	11	2	Gain	4.24E-02
MIR935	3	15	10	7	11	2	Gain	4.24E-02
MYADM	3	15	10	7	11	2	Gain	4.24E-02
PRKCG	3	15	10	7	11	2	Gain	4.24E-02
VSTM1	3	15	10	7	11	2	Gain	4.24E-02
ZNF528	3	15	10	7	11	2	Gain	4.24E-02
ZNF534	3	15	10	7	11	2	Gain	4.24E-02
ZNF578	3	15	10	7	11	2	Gain	4.24E-02
ZNF610	3	15	10	7	11	2	Gain	4.24E-02
ZNF808	3	15	10	7	11	2	Gain	4.24E-02
ZNF880	3	15	10	7	11	2	Gain	4.24E-02
COL13A1	9	13	6	0	14	6	Loss	4.12E-03
ACSF3	17	7	4	5	11	4	Loss	1.48E-02
ANKRD11	17	7	4	5	11	4	Loss	1.48E-02
CDH15	17	7	4	5	11	4	Loss	1.48E-02
NCRNA00304	17	7	4	5	11	4	Loss	1.48E-02
ZNF778	17	7	4	5	11	4	Loss	1.48E-02
ASCC1	9	15	4	1	15	4	Loss	2.31E-02
CHST3	9	15	4	1	15	4	Loss	2.31E-02
SPOCK2	9	15	4	1	15	4	Loss	2.31E-02
ANPEP	6	19	3	0	13	7	Loss	3.07E-02
AP3S2	6	19	3	0	13	7	Loss	3.07E-02
C15orf38	6	19	3	0	13	7	Loss	3.07E-02
C15orf38-AP3S2	6	19	3	0	13	7	Loss	3.07E-02
C15orf42	6	19	3	0	13	7	Loss	3.07E-02
KIF7	6	19	3	0	13	7	Loss	3.07E-02
LOC283761	6	19	3	0	13	7	Loss	3.07E-02
MAN2A2	6	19	3	0	10	10	Loss	3.07E-02
MESP1	6	19	3	0	13	7	Loss	3.07E-02
MESP2	6	19	3	0	13	7	Loss	3.07E-02
PEX11A	6	19	3	0	13	7	Loss	3.07E-02
PLIN1	6	19	3	0	13	7	Loss	3.07E-02
PRC1	6	19	3	0	10	10	Loss	3.07E-02
RCCD1	6	19	3	0	10	10	Loss	3.07E-02
RHCG	6	19	3	0	13	7	Loss	3.07E-02
VPS33B	6	19	3	0	10	10	Loss	3.07E-02
WDR93	6	19	3	0	13	7	Loss	3.07E-02
ZNF710	6	19	3	0	13	7	Loss	3.07E-02
CCAR1	8	16	4	1	16	3	Loss	4.12E-02
SNORD98	8	16	4	1	16	3	Loss	4.12E-02
TET1	8	16	4	1	16	3	Loss	4.12E-02

Table S18. Alteration statistics in gastric cancers.

	Sample	# of All Somatic SNVs	# of Somatic SNVs in CDS	# of Somatic Indels in CDS	# of genes affected by somatic CDS Indels And nsSNVs	# of Somatic CNVs	Somatic CNV (Loss and Gain) Size (bp)	# of Genes Affected by Somatic CNVs	Somatic Copy Loss Size (bp)	# of Genes Affected by Somatic Copy Loss	Somatic Copy Gain Size (bp)	# of Genes Affected by Somatic Copy Gain	# of Somatic SVs	# of Genes Affected by Somatic SV breakpoints
Diffuse Type	D-01T	10,620	60	3	47	77	488,575,000	4,532	245,654,000	1,911	242,921,000	2,626	675	213
	D-02T	6,736	33	2	30	102	809,875,000	9,320	510,641,000	8,079	299,234,000	1,243	460	101
	D-03T	27,797	56	-	40	10	24,248,000	23	21,113,000	-	3,135,000	23	414	209
	D-04T	10,230	44	3	43	42	487,697,000	4,242	303,126,000	3,560	184,571,000	682	421	38
	D-05T	8,946	48	5	44	66	779,205,000	6,286	442,281,000	2,724	336,924,000	3,564	503	53
	D-06T	7,130	23	1	23	3	21,000	-	4,000	-	17,000	-	342	49
	D-07T	16,895	94	1	78	10	275,479,000	1,262	68,970,000	133	206,509,000	1,129	649	194
	D-08T	8,972	8	1	9	2	1,589,000	26	-	-	1,589,000	26	537	570
	D-09T	11,515	33	-	30	15	244,458,000	1,509	180,611,000	460	63,847,000	1,049	694	310
	D-10T	19,473	109	4	88	108	1,054,714,000	9,777	685,280,000	7,563	369,434,000	2,216	619	62
	D-11T	8,753	60	1	48	91	996,645,000	10,095	686,598,000	8,857	310,047,000	1,238	318	40
	D-12T	12,410	84	2	76	10	222,734,000	1,922	81,578,000	1,014	141,156,000	908	679	49
	D-13T	7,792	49	1	43	285	1,489,548,000	13,603	955,860,000	12,208	533,688,000	1,409	782	195
	D-14T	14,703	58	1	48	34	255,209,000	3,660	254,541,000	3,660	668,000	-	640	216
Intestinal Type	I-01T	28,904	160	4	116	90	1,971,350,000	15,193	1,697,815,000	13,811	273,535,000	1,388	1,137	198
	I-02T	36,931	131	2	111	60	849,219,000	6,712	443,732,000	4,193	405,487,000	2,520	688	158
	I-03T	15,879	58	1	52	12	148,436,000	1,958	25,882,000	587	122,554,000	1,371	397	114
	I-04T	9,430	23	-	21	27	318,843,000	3,872	222,185,000	3,661	96,658,000	211	429	89
Mean	All Samples	14,618	63	2	53	58	578,769,167	5,222	379,215,056	4,023	199,554,111	1,200	577	159
	Diffuse Type	12,284	54	2	46	61	509,285,500	4,733	316,875,500	3,584	192,410,000	1,151	552	164
	Intestinal Type	22,786	93	2	75	47	821,962,000	6,934	597,403,500	5,563	224,558,500	1,373	663	140
P-value*		2.53.E-02	8.23.E-02	9.68.E-01	8.91.E-02	7.29.E-01	3.25.E-01	4.23.E-01	2.60.E-01	4.37.E-01	7.32.E-01	7.12.E-01	3.38.E-01	7.51.E-01

*P-value by t-test between diffuse and intestinal types

Table S19. Overview of genetic variations in 18 gastric cancer samples.

Gene	Diffuse-type														Intestinal-type			
	D-01T	D-02T	D-03T	D-04T	D-05T	D-06T	D-07T	D-08T	D-09T	D-10T	D-11T	D-12T	D-13T	D-14T	I-01T	I-02T	I-03T	I-04T
CDH1	Red	N, Red	S	Red	NI				NV	Red	Red	N	V, Red	V	Red	Red	NV	Red
MYC	Blue	Blue			Blue		Blue				Blue							
ZHX2		Blue			Blue		Blue				Blue							
MOS	Blue	Blue					Blue			Blue	Blue				Red			
MDM2	Blue	Blue								Blue					Red			
MET	Red	Blue		Blue							Blue							
PIK3CA		N	V	N, Blue		N	Blue			Blue	N	N					Blue	
TP53		Red		Red	Red					N, Red	Red		N, Red	Red	Red	N, Red		S, Red
ARID1A	Red	Red		Red	Red		V	N		V, Red	Red		Red	Red	Red		V	Red
RUNX3	Red	Red		Red	Red					Red	Red		Red	Red	Red			Red
PLA2G2A	Red	Red		Red	Red					Red	Red		Red	Red	Red			Red
SMAD2		Red							Red	Red		N	Red		Red			
FHIT	V						V	V	V	V, Red	V, Red		V	V, Red		V, Red	V	V
WWOX	Red	V					V				Red		Red	V, Red	Red	Red	V	V
MIPOL1								V						V, Red	Red		V, Blue	
PTEN					V, Red				V	D					V, Red		Blue	
TSC2	Blue	Red		Red	Blue					Red	Red		Red	Red			V, Blue	Red
RNF216		Red			Blue					Red			Red	Red	Red		V	Red
PSCA	Blue				Blue		Blue			Red			Red					Red
CACNG6					Blue				Blue		Red		Red	Red	Red			
CACNG7					Blue				Blue		Red		Red	Red	Red			
CACNG8					Blue				Blue		Red		Red	Red	Red			

N = nsSNV, S = splicing site SNV, I = small insertion, D = small deletion, V = SV breakpoint, Red Box = copy number loss, Blue Box = copy number gain

Table S20. Nonsense mutations in *CDH1* found in diffuse gastric cancers.

a. Somatic mutations in this study and previously reported exome data

Mutation	in silico function prediction	Sample	Evidences
N256S	loss	D-02	EC1-2 junction, adjacent residue from calcium binding site
Splicing site	loss	D-03	Splicing site mutation
D257N	loss	D-05	EC1-2 junction, adjacent residue from calcium binding site
S829fs	loss	D-05	Frame shift
V252G	loss	D-09	EC1's beta barrel, nonpolar (hydrophobic) -> non hydrophobic
Q23*	loss	D-12	Pre maturation
D221G	loss	I-03	EC1-2 junction, adjacent residue from calcium binding site
P127fs	loss	Diffuse (Wang *et al*.)	Frame shift
V694I	functional	MSI intestinal (Wang *et al*.)	Loop region between EC5 and a transmembrane region

b. Mutations in hereditary diffuse-type gastric cancer from previously studies

Mutation	in vitro function	Evidences
T118R	loss	no structure, propeptide
E185V	functional	EC1's surface exposed loop region, not dimerization part
L214P	loss	EC1's beta barrel, nonpolar (hydrophobic) -> polar
S232C	functional	EC1's beta-barrel, similar side chain
G239R	loss	EC1's surface exposed loop region, maybe cis-dimerization part
G274S	functional	EC2-3 junction, distant residue from calcium binding site
A298T	loss	EC1-2 junction, adjacent residue from calcium binding site
T340A	loss	EC2's beta-barrel, polar -> nonpolar
P373L	loss	EC2-3 junction, adjacent residue from calcium binding site
W409R	loss	EC2-3 junction, adjacent residue from calcium binding site
P429S	loss	EC3's beta-barrel loop, hydrophobic -> polar
L583R	loss	EC4's beta-barrel, nonpolar -> positively charged
L583I	functional	EC4's beta-barrel, similar side chain
A592T	functional	EC4-5 junction
A617T	functional	EC4-5 junction
A634V	loss	EC5's beta-barrel loop, similar side chain but different in size
R732Q	loss	catenin binding domain
P799R	loss	catenin binding domain
V832M	loss	catenin binding domain
